# Gefitinib Plus Chemotherapy vs Gefitinib Alone in Untreated *EGFR*-Mutant Non–Small Cell Lung Cancer in Patients With Brain Metastases: The GAP BRAIN Open-Label, Randomized, Multicenter, Phase 3 Study

**DOI:** 10.1001/jamanetworkopen.2022.55050

**Published:** 2023-02-08

**Authors:** Xue Hou, Meichen Li, Guowu Wu, Weineng Feng, Jin Su, Honghua Jiang, Guanming Jiang, Jing Chen, Baishen Zhang, Zhixuan You, Qing Liu, Likun Chen

**Affiliations:** 1 State Key Laboratory of Oncology in South China, Collaborative Innovation Center for Cancer Medicine, Department of Medical Oncology, Sun Yat-Sen University Cancer Center, Guangzhou, China; 2 Cancer Center, Department of Medical Oncology, Meizhou People’s Hospital, Meizhou, China; 3 Department of Head and Neck/Thoracic Medical Oncology, the First People's Hospital of Foshan, Foshan, China; 4 Department of Respiratory and Critical Care Medicine, Chronic Airways Diseases Laboratory, Nanfang Hospital, Southern Medical University, Guangzhou, China; 5 Department of Oncology, Southern Theater Air Force Hospital, Guangzhou, China; 6 Dongguan Institute of Clinical Cancer Research, Department of Medical Oncology, Southern Medical University-affiliated Dongguan People's Hospital, Dongguan, China; 7 State Key Laboratory of Respiratory Disease, National Clinical Research Center for Respiratory Disease, Guangzhou Institute of Respiratory Disease, the First Affiliated Hospital of Guangzhou Medical University, Guangzhou, China; 8 State Key Laboratory of Oncology in South China, Collaborative Innovation Center for Cancer Medicine, Department of Statistics, Sun Yat-Sen University Cancer Center, Guangzhou, China

## Abstract

**Importance:**

Use of tyrosine kinase inhibitors (TKIs) is the standard therapy for epidermal growth factor receptor (*EGFR*)–mutated non–small cell lung cancer (NSCLC) with brain metastases. Several studies have shown that adding chemotherapy to EGFR-TKIs could improve progression-free survival (PFS) in patients with *EGFR*-mutant advanced NSCLC; however, the efficacy of these agents in patients with brain metastases remains unclear.

**Objective:**

To investigate the efficacy and safety of gefitinib plus chemotherapy (pemetrexed with platinum) compared with gefitinib alone in patients with untreated *EGFR-*mutant NSCLC brain metastases.

**Design, Setting, and Participants:**

This open-label prospective, multicenter, phase 3 randomized clinical trial was conducted in 6 centers in China from January 13, 2016, to August 27, 2021. The median follow-up time was 21.1 months (IQR, 13.5-31.8 months). Patients with untreated confirmed brain metastases and *EGFR*-sensitive mutated NSCLC were enrolled.

**Interventions:**

The eligible patients were randomly assigned (1:1) to receive gefitinib plus chemotherapy or gefitinib alone.

**Main Outcomes and Measures:**

The primary end point was intracranial PFS; secondary end points included PFS, overall survival (OS), intracranial objective response rate, overall objective response rate, and safety. Intention-to-treat analysis was performed.

**Results:**

A total of 161 patients (87 [54.0%] women; mean [SD] age, 55 [9.8] years; range, 26-80 years) were enrolled and randomized to receive gefitinib (n = 81) or gefitinib plus chemotherapy (n = 80). The median intracranial PFS was 15.6 months (95% CI, 14.3-16.9 months) in the gefitinib plus chemotherapy group vs 9.1 months (95% CI, 8.0-10.2 months) in the gefitinib group (hazard ratio, 0.36; 95% CI, 0.25-0.53; *P* < .001). Similarly, the median PFS was significantly longer with gefitinib plus chemotherapy than gefitinib alone (16.3; 95% CI, 14.4-18.2 months vs 9.5; 95% CI, 8.3-10.8 months; *P* < .001). Gefitinib plus chemotherapy had a better intracranial objective response rate (85.0%; 95% CI, 77.0%-93.0% vs 63.0%; 95% CI, 52.2%-73.7%; *P* = .002) and overall objective response rate (80.0%; 95% CI, 71.0%-89.0% vs 64.2%; 95% CI, 53.5%-74.9%; *P* = .03) than gefitinib alone. At data cutoff, the median OS was also significantly longer in the gefitinib plus chemotherapy group vs the gefitinib group (35.0 vs 28.9 months; hazard ratio, 0.65; 95% CI, 0.43-0.99; *P* = .04). Grade 3 or worse adverse events were more common with gefitinib plus chemotherapy, most of which were manageable.

**Conclusions and Relevance:**

In this randomized clinical trial, gefitinib plus chemotherapy significantly improved intracranial PFS, PFS, and OS compared with gefitinib alone in patients with untreated *EGFR-*mutant NSCLC brain metastases and could be an optional first-line treatment for these patients.

**Trial Registration:**

ClinicalTrials.gov Identifier: NCT01951469

## Key Points

**Question:**

What are the efficacy and safety of gefitinib plus chemotherapy in patients with untreated epidermal growth factor receptor (*EGFR*) mutated non–small cell lung cancer (NSCLC) brain metastases?

**Findings:**

In this randomized clinical trial including 161 patients with untreated *EGFR*-mutant NSCLC brain metastases, gefitinib plus chemotherapy significantly improved intracranial progression-free survival, overall progression-free survival, and overall survival than gefitinib alone, with manageable adverse events. Gefitinib plus chemotherapy also had better intracranial, extracranial, and overall response rates than gefitinib alone.

**Meaning:**

The findings of this randomized clinical trial suggest that gefitinib plus chemotherapy may be a viable first-line treatment for patients with brain metastases associated with *EGFR*-mutant NSCLC.

## Teaser

This randomized clinical trial compares the use of gefitinib plus chemotherapy with gefitinib monotherapy in patients with untreated *EGFR*-mutant non–small cell lung cancer brain metastases.

## Introduction

Brain metastases occur in approximately 30% to 40% of patients with non–small cell lung cancer (NSCLC) during the course of the disease and 20% to 25% of patients with advanced NSCLC have brain metastasis at the initial diagnosis.^1^ Patients with epidermal growth factor receptor (*EGFR*)-mutant NSCLC were more prone to the development of brain metastases, with an approximate frequency of 44% to 63% during the treatment course,^2,3,4^ which is higher than in patients with *EGFR* wild-type. Improving the treatment outcome of patients with brain metastases became the key point of management of treatment for patients with *EGFR*-mutant NSCLC.

Historically, brain metastases were treated with surgical resection, radiotherapy, and antitumor agents, either alone or in combination. For patients with metastatic NSCLC harboring *EGFR* mutation, *EGFR* tyrosine kinase inhibitors (TKIs) have been the standard first-line treatment,^5,6^ and accumulating evidence suggests EGFR-TKIs also exhibit efficacy on intracranial lesions.^7,8,9^ A phase 3 study (BRAIN) demonstrated that patients who received the first-generation EGFR-TKI icotinib have significantly longer intracranial progression-free survival (PFS) and fewer adverse events than those who received whole-brain irradiation plus chemotherapy, indicating the EGFR-TKIs were better first-line treatment in patients with *EGFR-*mutant NSCLC brain metastases.^10^

Although superior efficacy of EGFR-TKIs was shown, resistance to treatment with the first-generation EGFR-TKIs developed, and their median PFS is approximately 8 to 12 months. Several strategies have been explored to improve PFS and overcome resistance, including use of next-generation EGFR-TKIs and EGFR-TKI combination treatment. The third-generation EGFR-TKI osimertinib prolonged PFS to 18 months as first-line treatment and to approximately 15 months in a subgroup of patients with brain metastases. The vascular endothelial growth factor signaling pathway is a candidate target for combination therapy; however, the efficacy of vascular endothelial growth factor treatment in patients with brain metastasis is controversial.^11,12^ Treatment with EGFR-TKIs combined with chemotherapy was another strategy. In early clinical trials, adding EGFR-TKIs to chemotherapy showed no significant improvement of PFS, partially because patients were not selected for *EGFR*-sensitive mutation.^13,14,15^ However, clinical trials showed that the combination of chemotherapy and gefitinib could significantly improve PFS in patients with *EGFR*-mutant NSCLC, which makes a case for revisiting the combination therapy strategy.^16,17,18^ The NEJ009 Study demonstrated superior PFS benefit in a subgroup analysis of patients with brain metastases,^17^supplying a promising strategy for these patients. However, the statistical deficiency and sample size of subgroup analysis limited the generalization of the conclusion, and prospective randomized trials for patients with *EGFR*-mutant brain metastases are urgently required. Herein, we report the results of a phase 3 trial that compared gefitinib plus pemetrexed with platinum (chemotherapy) with gefitinib alone for the first-line treatment in patients with asymptomatic *EGFR*-mutant NSCLC brain metastases.

## Methods

### Study Design and Participants

This was an open-label, parallel, phase 3 randomized clinical trial (GAP BRAIN) conducted in 6 centers in China. The main eligibility criteria included histologically or cytologically confirmed NSCLC with *EGFR*-sensitive mutation (exon 19 deletion or exon 21 L858R mutation); confirmed brain metastases noted on enhanced brain magnetic resonance imaging; asymptomatic brain metastases; at least 3 intracranial metastatic lesions or patients with 1 to 2 intracranial lesions who are not suitable for localized treatment or refused to receive localized treatment for intracranial metastatic lesions; at least 1 intracranial evaluable lesion, which was defined as lesions with the longest diameter of greater than 5 mm according to modified Response Evaluation Criteria in Solid Tumours [RECIST] 1.1 guidelines (based on 1 mm for magnetic resonance imaging scan slices)^19,20^; treatment naive; aged 18 to 80 years; Eastern Cooperative Oncology Group (ECOG) performance status of 0 or 1; adequate organ function; and a life expectancy of 12 weeks or more. The main exclusion criteria were previous systemic or localized therapy; obvious central nervous system symptoms and lack of response to treatment of dehydration; radiologically or pathologically confirmed leptomeningeal metastases; history of interstitial lung disease, radiation-associated pneumonitis that required corticosteroid treatment, or any evidence of clinical active interstitial lung disease; concomitant serious systemic disorders; and any second primary malignant disease within 5 years. This study was performed according to the Declaration of Helsinki,^21^ and also reported following the Consolidated Standards of Reporting Trials (CONSORT) reporting guideline. The study protocol was approved by the ethics committees of Sun Yat-Sen University Cancer Center and other participating centers. The study protocol and statistical analysis plan are available in Supplement 1. All patients provided written informed consent; participants did not receive financial compensation.

### Randomization

The eligible patients were randomly assigned (1:1) to receive gefitinib plus pemetrexed with platinum chemotherapy or gefitinib alone. Random assignment was performed using a computer-generated randomization sequence. The Clinical Trials Center of Sun Yat-Sen University Cancer Center generated the randomization sequence, confirmed participant eligibility, assigned the eligible patients to trial groups, and notified investigators of treatment allocation for each patient. Patients and investigators were not blinded to treatment allocation.

### Procedure

Patients assigned to the gefitinib-alone group received gefitinib, 250 mg, once daily; patients assigned to the gefitinib plus chemotherapy group received gefitinib, 250 mg, once daily with chemotherapy (pemetrexed, 500 mg/m^2^, combined with cisplatin, 75 mg/m^2^, or nedaplatin, 80 mg/m^2^, in a 4-week cycle for 4 to 6 cycles, followed by pemetrexed, 500 mg/m^2^, as maintenance every 4 weeks). Patients continued treatment until disease progression, development of unacceptable adverse events, or any cause of death. Patients were allowed to receive granulocyte colony-stimulating factor, antiemetics, and other supportive treatment. After disease progression, subsequent treatment was at the discretion of the physician.

Tumor evaluation for intracranial and extracranial lesions was independently assessed by investigators. The number of intracranial target lesions was extended to 5 lesions, as well as 5 extracranial target lesions according to modified RECIST 1.1 guidelines. Tumor assessments were performed within the 3 weeks before enrollment, then every 8 weeks (enhanced computed tomography scans for extracranial lesions and enhanced magnetic resonance imaging for intracranial lesions) until disease progression was noted. Physical and laboratory examinations were performed within 7 days before enrollment, every 4 weeks during treatment, and at the time of disease progression. After disease progression, follow-up for survival analysis was performed every 3 months. All adverse events were evaluated according to the National Cancer Institute Common Terminology Criteria for Adverse Events, version 4.0.^22^

### Outcomes

The primary end point was intracranial PFS, defined as time from randomization to intracranial disease progression or death. The secondary end points included PFS, defined as time from randomization to overall disease (both intracranial and extracranial) progression or death; overall survival (OS), defined as time from randomization to death from any cause; intracranial objective response rate, defined as proportion of patients with complete or partial response of intracranial lesions; objective response rate, defined as proportion of patients with complete or partial response of overall lesions; and safety.

### Statistical Analysis

We hypothesized that median intracranial PFS be prolonged from 8 months in the gefitinib-alone group to 12 months in the gefitinib plus chemotherapy group. Assuming a hazard ratio (HR) of 0.67 for gefitinib plus chemotherapy vs gefitinib alone, with 80% power and 1-sided α value of 10%, our estimated sample size was 160 patients.

Intention-to-treat analysis was performed. Intracranial PFS, PFS, and OS were estimated using Kaplan-Meier curves, and differences between groups were compared with a stratified log-rank test. Hazard ratios and 95% CIs were evaluated with Cox proportional hazards regression models. Tumor overall objective response rate, disease control rate, and incidence of adverse events were compared with the Fisher exact test between the 2 treatment groups. All end points are reported as 2-sided *P* values in this study, with a significance threshold of .05. All statistical analyses were performed using R software, version 4.0.5 (R Foundation for Statistical Computing).

## Results

### Patients

From January 13, 2016, to August 27, 2021, a total of 161 patients (87 [54.0%] women, 74 [46.0%] men; mean [SD] age, 55 [9.8] years; range, 26-80 years) were enrolled and randomized to receive gefitinib (n = 81) or gefitinib plus chemotherapy (n = 80) (eFigure 1 in Supplement 2). All randomized patients received at least 1 dose of study drugs and were included in efficacy and safety analyses. The baseline characteristics were balanced between the 2 groups (Table 1). Most patients were nonsmokers and had lung adenocarcinoma. Regarding *EGFR* mutation type, 85 patients (52.8%) had exon 19 deletions, 70 (43.5%) had exon 21 L858R mutations, and 6 (3.7%) had uncommon *EGFR* mutations. In addition, 37 patients (45.7%) in the gefitinib group and 47 patients (58.7%) in the gefitinib plus chemotherapy group had baseline next-generation sequencing data with *TP53* mutation type. At the data cutoff, the median follow-up time was 21.1 months (IQR, 13.5-31.8 months); 18 patients in the gefitinib plus chemotherapy group and 10 patients in the gefitinib group were still receiving study treatment.

**Table 1.** Baseline Characteristics of the Study Population

Characteristics	Patients, No. (%)		
	Total (N = 161)	Gefitinib (n = 81)	Gefitinib plus chemotherapy (n = 80)^a^
Age, median (range), y	55 (26-80)	56 (26-80)	55 (34-72)
Sex			
Male	74 (46.0)	38 (46.9)	36 (45.0)
Female	87 (54.0)	43 (53.1)	44 (55.0)
Smoking status			
Smoker	41 (25.5)	21 (25.9)	20 (25.0)
Nonsmoker	120 (74.5)	60 (74.1)	60 (75.0)
ECOG performance status			
0	43 (26.7)	19 (23.5)	24 (30.0)
1	118 (73.3)	62 (76.5)	56 (70.0)
Histologic characteristics			
Adenocarcinoma	153 (95.0)	77 (95.1)	76 (95.0)
Other^b^	8 (5.0)	4 (4.9)	4 (5.0)
*EGFR* mutation types			
Exon 19 deletion	85 (52.8)	45 (55.6)	40 (50.0)
Exon 21 L858R	70 (43.5)	35 (43.2)	35 (43.7)
Uncommon mutation^c^	6 (3.7)	1 (1.2)	5 (6.3)
Extracranial metastases			
Pleura	39 (24.2)	23 (28.4)	16 (20.0)
Bone	101 (62.7)	50 (61.7)	51 (63.8)
Liver	15 (9.3)	9 (11.1)	6 (7.5)
Adrenal gland	30 (18.6)	15 (18.5)	15 (18.7)
Intracranial tumor, No.^d^			
1-3	63 (39.1)	35 (43.2)	28 (35.0)
≥4	98 (60.9)	46 (56.8)	52 (65.0)
Intracranial tumor size, mm^d^			
<20	110 (68.3)	57 (70.4)	53 (66.3)
≥20	51 (31.7)	24 (29.6)	27 (33.7)
Lung-molGPA^e^			
1.5-2	15 (9.3)	6 (7.4)	9 (11.2)
2.5-3	101 (62.7)	47 (58.0)	54 (67.5)
3.5-4	45 (28.0)	28 (34.6)	17 (21.2)
*TP53* mutation, No.^f^	84	37	47
Yes	62 (73.8)	25 (67.6)	37 (78.7)
No	22 (26.2)	12 (32.4)	10 (21.3)
Chemotherapy regimen			
Pemetrexed/cisplatin	33 (20.5)	NA	33 (41.3)
Pemetrexed/nedaplatin	47 (29.2)	NA	47 (58.7)

Abbreviations: DS-GPA, diagnosis-specific Graded Prognostic Assessment; ECOG, Eastern Cooperative Oncology Group; *EGFR*, epidermal growth factor receptor; lung-molGPA, graded prognostic assessment for lung cancer with brain metastases using molecular markers; NA, not applicable.

^a^ Chemotherapy comprised pemetrexed, 500 mg/m^2^, combined with cisplatin, 75 mg/m^2^, or nedaplatin, 80 mg/m^2^, in a 4-week cycle for 4 to 6 cycles, followed by pemetrexed, 500 mg/m^2^, as maintenance every 4 weeks.

^b^ Includes 4 patients with poorly differentiated non–small cell lung cancer not further specified, 2 patients with adenosquamous cell carcinoma, 1 patient with squamous cell carcinoma, and 1 patient with sarcomatoid carcinoma.

^c^ Includes 3 patients with *EGFR* L861Q, 2 patients with *EGFR* G719X, and 1 patient with *EGFR* G719A.

^d^ Intracranial tumor characteristics were evaluated according to baseline-enhanced brain magnetic resonance imaging.

^e^ An update of the DS-GPA using molecular markers.

^f^ Eighty-four patients had baseline next-generation sequencing data for *TP53* mutation.

### Intracranial Efficacy

**Figure 1.**
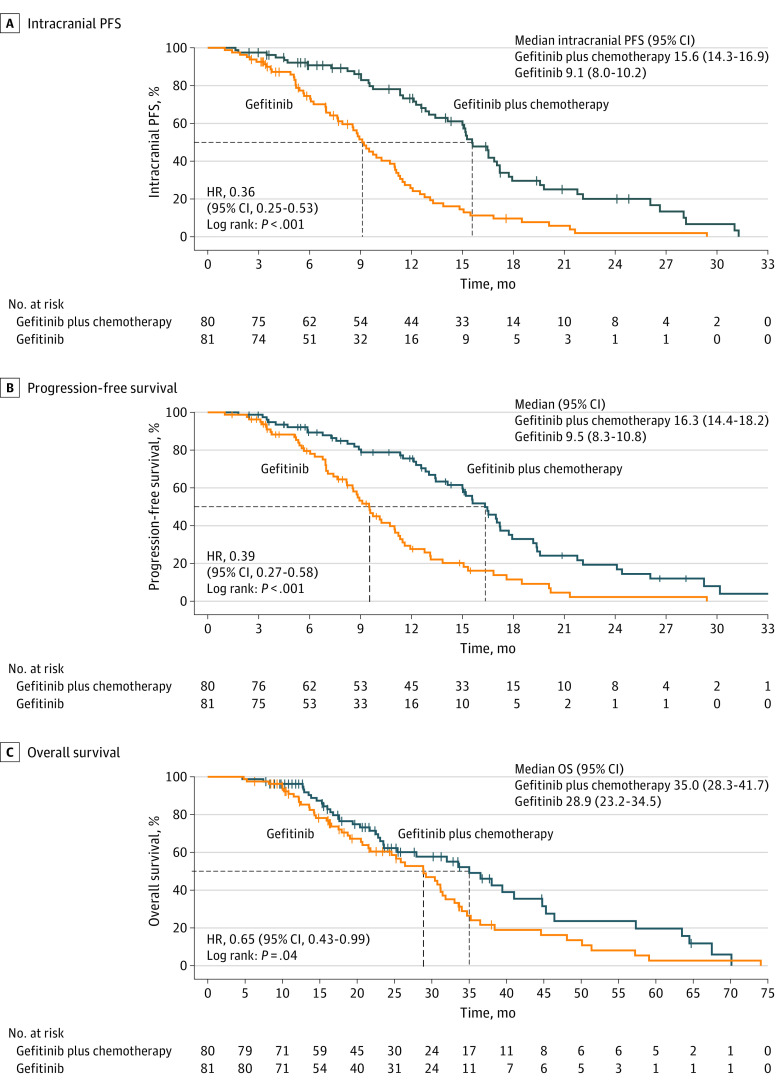
Estimates of Outcomes in the Intention-to-Treat Population Findings shown for intracranial progression-free survival (PFS) (A), PFS (B), and overall survival (OS) (C). Chemotherapy comprised pemetrexed, 500 mg/m^2^, combined with cisplatin, 75 mg/m^2^, or nedaplatin, 80 mg/m^2^, in a 4-week cycle for 4 to 6 cycles, followed by pemetrexed, 500 mg/m^2^, as maintenance every 4 weeks. *P* values were calculated using a stratified log-rank test. HR indicates hazard ratio.

**Figure 2.**
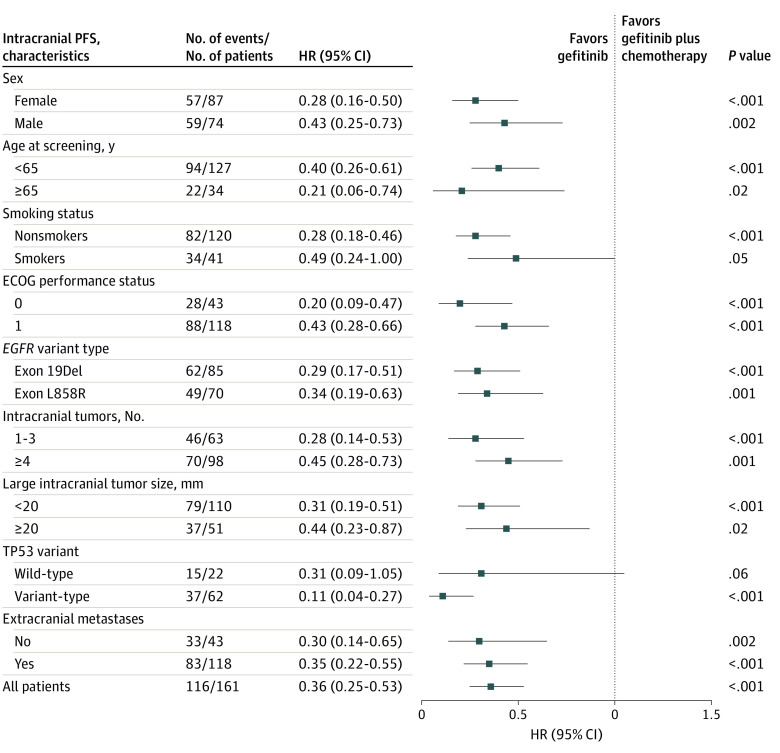
Analysis of Intracranial Progression-Free (PFS) Survival Hazard ratios (HRs) and corresponding 95% CIs were evaluated using Cox proportional hazards regression model. Chemotherapy comprised pemetrexed, 500 mg/m^2^, combined with cisplatin, 75 mg/m^2^, or nedaplatin, 80 mg/m^2^, in a 4-week cycle for 4 to 6 cycles, followed by pemetrexed, 500 mg/m^2^, as maintenance every 4 weeks. ECOG indicates Eastern Cooperative Oncology Group; Del, deletion; and *EGFR*, epidermal growth factor receptor.

At the data cutoff, 51 patients (63.8%) in the gefitinib plus chemotherapy group and 65 patients (80.2%) in the gefitinib group had confirmed intracranial disease progression. The median intracranial PFS was 15.6 months (95% CI, 14.3-16.9 months) in the gefitinib plus chemotherapy group vs 9.1 months (95% CI, 8.0-10.2 months) in the gefitinib group (HR, 0.36; 95% CI, 0.25-0.53; *P* < .001) (Figure 1A). In subgroup analysis based on baseline characteristics, intracranial PFS favored gefitinib plus chemotherapy over gefitinib in most subgroups (Figure 2), whereas the benefit was not statistically significant in the subgroup of patients with *TP53* wild-type. Similarly, the gefitinib plus chemotherapy group achieved a better intracranial objective response rate than the gefitinib group (85.0%, 95% CI, 77.0%-93.0% vs 63.0%; 95% CI, 52.2%-73.7%; *P* = .002); odds ratios are given in Table 2. The maximum tumor change from baseline in intracranial tumors is shown in eFigure 3 in Supplement 2.

**Table 2.** Tumor Response in the Intention-to-Treat Population^a^

Variable	Intracranial response				Extracranial response					Overall response			
	No. (%)		OR (95% CI)	*P* value	No. (%)		OR (95% CI)	*P* value	No. (%)			OR (95% CI)	*P* value
	Gefitinib plus chemotherapy^b^	Gefitinib			Gefitinib plus chemotherapy^b^	Gefitinib			Gefitinib plus chemotherapy^b^		Gefitinib		
Response													
Complete	9 (11.3)	9 (11.1)	NA	NA	0	0	NA	NA	0		0	NA	NA
Partial	59 (73.7)	42 (51.8)	NA	NA	61 (76.3)	47 (58.0)	NA	NA	64 (80.0)		52 (64.2)	NA	NA
Disease													
Stable	8 (10.0)	25 (30.9)	NA	NA	17 (21.2)	30 (37.1)	NA	NA	12 (15.0)		25 (30.9)	NA	NA
Progressive	2 (2.5)	3 (3.7)	NA	NA	0	1 (1.2)	NA	NA	2 (2.5)		1 (1.2)	NA	NA
Not available	2 (2.5)	2 (2.5)	NA	NA	2 (2.5)	3 (3.7)	NA	NA	2 (2.5)		3 (3.7)	NA	NA
Objective response rate	68 (85.0)	51 (63.0)	3.33 (1.56-7.14)	.002	61 (76.3)	47 (58.0)	2.32 (1.18-4.58)	.02	64 (80.0)		52 (64.2)	2.07 (1.02-4.17)	.04
Disease control rate	76 (95.0)	76 (93.8)	1.25 (0.32-4.84)	>.99	78 (97.5)	77 (95.1)	2.03 (0.36-11.37)	.68	76 (95.0)		77 (95.1)	0.99 (0.24-4.09)	>.99

Abbreviations: NA, not applicable; OR, odds ratio.

^a^ Responses were evaluated according to the Response Evaluation Criteria in Solid Tumours 1.1 guideline. *P* values were assessed using the Fisher exact test. Odds ratios and 95% CIs were assessed using the logistic regression model.

^b^ Chemotherapy comprised pemetrexed, 500 mg/m^2^, combined with cisplatin, 75 mg/m^2^, or nedaplatin, 80 mg/m^2^, in a 4-week cycle for 4 to 6 cycles, followed by pemetrexed, 500 mg/m^2^, as maintenance every 4 weeks.

At disease progression, 50 patients had intracranial lesions progression only, 18 patients had extracranial lesions progression only, and 63 patients had simultaneous intracranial and extracranial lesions progression. Patients’ progressive patterns between the 2 groups is noted in eTable 1 in Supplement 2.

### Systemic Efficacy

At the data cutoff, 50 patients (62.5%) in the gefitinib plus chemotherapy group and 60 patients (74.1%) in the gefitinib group had systemic disease progression. The median PFS was 16.3 months (95% CI, 14.4-18.2 months) in the gefitinib plus chemotherapy group vs 9.5 months (95% CI, 8.3-10.8 months) in the gefitinib group (HR, 0.39; 95% CI, 0.27-0.58; *P* < .001) (Figure 1B). The overall objective response rate was significantly higher in the gefitinib plus chemotherapy group than in the gefitinib group (80.0%; 95% CI, 71.0%-89.0% vs 64.2%; 95% CI, 53.5%-74.9%; *P* = .03) (Table 2); subgroup analyses also demonstrated gefitinib plus chemotherapy obtained PFS benefit in most subgroups (eFigure 2 in Supplement 2).

**Figure 3.**
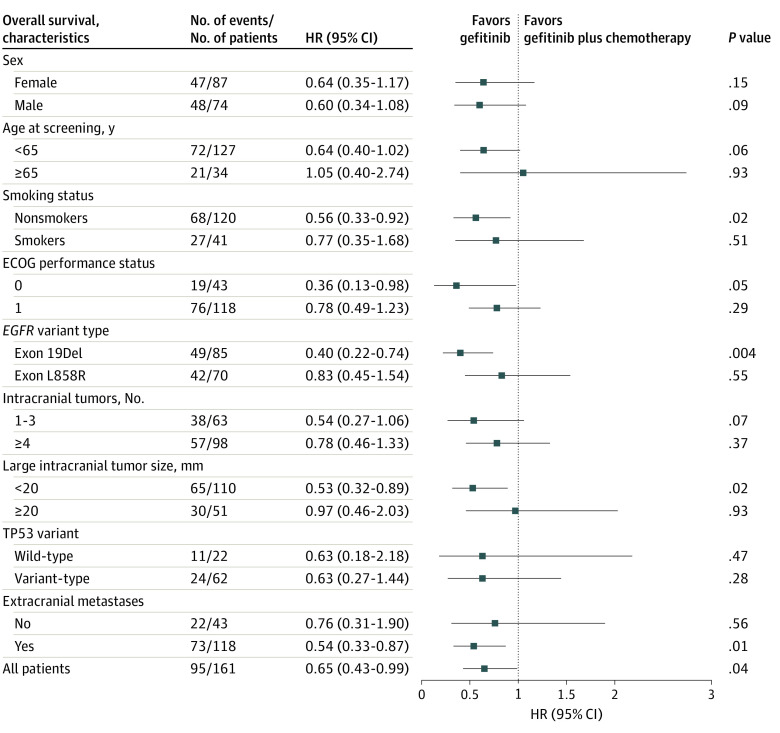
Analysis of Overall Survival Hazard ratios (HRs) and corresponding 95% CIs were evaluated using Cox proportional hazards regression model. Chemotherapy comprised pemetrexed, 500 mg/m^2^, combined with cisplatin, 75 mg/m^2^, or nedaplatin, 80 mg/m^2^, in a 4-week cycle for 4 to 6 cycles, followed by pemetrexed, 500 mg/m^2^, as maintenance every 4 weeks. ECOG indicates Eastern Cooperative Oncology Group; Del, deletion; and *EGFR*, epidermal growth factor receptor.

At the data cutoff, 59.0% of patients (41 patients in the gefitinib plus chemotherapy group and 54 patients in the gefitinib group) had died. The median OS was significantly longer in the gefitinib plus chemotherapy group than in the gefitinib group (35.0; 95% CI, 28.3-41.7 vs 28.9; 95% CI, 23.2-34.5 months; HR, 0.65; 95% CI, 0.43-0.99; *P* = .04) (Figure 1C). The 3-year OS rate was 48.8% (95% CI, 37.6%-59.9%) in the gefitinib plus chemotherapy group and 24.1% (95% CI, 14.9%-33.9%) in the gefitinib group (*P* = .002). In subgroup analyses, the OS benefit of gefitinib plus chemotherapy was noted in subgroups of patients with *EGFR* 19del mutation, nonsmokers, good performance status, small intracranial lesions (largest diameter of intracranial lesion <20 mm), and patients with extracranial metastases (Figure 3).

### Safety Analysis

All 80 patients in the gefitinib plus chemotherapy group and 75 (92.6%) of the 81 patients in the gefitinib group experienced at least 1 drug-related adverse event. Of these, 32 patients (40.0%) in the gefitinib plus chemotherapy group and 17 patients (21.0%) in the gefitinib group reported grade 3 or worse adverse events (eTable 2 in Supplement 2). The most common grade 3 or worse adverse event was alanine aminotransferase level increase in both the gefitinib plus chemotherapy (9 [11.3%]) and gefitinib (12 [14.8%]) group. Ten patients (12.5%) in the gefitinib plus chemotherapy group and 7 patients (8.6%) in the gefitinib group experienced treatment interruption due to adverse events. One death due to pneumonitis occurred in the gefitinib plus chemotherapy group that was considered treatment related. No treatment-related deaths occurred in the gefitinib group (eTable 3 in Supplement 2).

### Postprogression Treatment

In total, 55 patients (68.8%) in the gefitinib plus chemotherapy group and 64 patients (79.0%) in the gefitinib group received at least 1 subsequent therapy after progression; details of the postprogression therapy are listed in eTable 4 in Supplement 2. At disease progression, *EGFR* Thr790Met was detected in 39 patients in the gefitinib plus chemotherapy group and 38 patients in the gefitinib group. Thirty-one of 77 patients (40.3%) overall developed *EGFR* T790M mutation (12 of 39 [30.8%] in the gefitinib plus chemotherapy group vs 19 of 38 [50.0%] in the gefitinib group), without a significant difference in this limited sample size (*P* = .11) (eFigure 4 in Supplement 2). Regarding second-line treatment, 6 patients in the gefitinib plus chemotherapy group and 8 patients in the gefitinib group received salvage brain radiotherapy (BRT), 32 patients in the gefitinib plus chemotherapy group and 31 patients in the gefitinib group received subsequent third-generation EGFR-TKIs as second-line treatment (per patient request in some who were *EGFR* T790M-negative). The PFS on subsequent third-generation EGFR-TKI use was not significantly different between the gefitinib plus chemotherapy vs gefitinib groups (7.7 vs 8.5 months; *P* = .75) (eFigure 5 in Supplement 2). Considering all subsequent treatment, 86 patients received third-generation EGFR-TKIs and 44 patients received BRT as second-line or further treatment. Patients who received third-generation EGFR-TKIs or BRT had longer OS than those who did not (eFigure 6 in Supplement 2). The median OS were 35.2 months in patients who received both TKIs and BRT, 28.8 months in those who received third-generation TKIs only, 22.8 months in those who received BRT only, and 16.4 months for patients who received neither third-generation TKIs nor BRT (*P* < .001) (eFigure 7 in Supplement 2).

## Discussion

In this phase 3 randomized clinical trial, our results revealed that gefitinib plus chemotherapy significantly improved intracranial PFS, PFS, and OS in patients with untreated NSCLC *EGFR* mutation and asymptomatic brain metastases. To our knowledge, this is the first randomized clinical trial to compare the intracranial efficacy and safety of gefitinib plus chemotherapy with gefitinib as first-line treatment in *EGFR*-mutant NSCLC with brain metastases.

Recently, EGFR-TKI combination therapy has shown superior efficacy than EGFR-TKI treatment alone in the NEJ009.^17^ Consistent with subgroup analysis of brain metastases in that study, our data showed that gefitinib plus chemotherapy has a similar magnitude of intracranial PFS benefit with overall PFS in patients with untreated brain metastases. Also, with all patients who had evaluable intracranial lesions, gefitinib plus chemotherapy showed better intracranial, extracranial, and overall response rates than gefitinib alone.

The third-generation EGFR-TKI osimertinib has demonstrated longer PFS and OS and better central nervous system permeability than first-generation EGFR-TKIs in the FLAURA study.^23,24^ At the start of our trial, the FLAURA study had not reported results, and first-generation EGFR-TKIs were still the standard first-line treatment for advanced *EGFR*-mutant NSCLC. However, for Asian patients and patients with brain metastases, the OS benefit was less with osimertinib compared with standard EGFR-TKI treatment in the global FLAURA and FLAURA China studies.^25,26^ Due to a complicated resistance mechanism, there are no standard targeted treatment options after disease progression while patients receive osimertinib—more than two-thirds of the patients could only receive cytotoxic chemotherapy and immunotherapy, which provided little benefit.^24^ In our study, the median intracranial PFS (15.6 months) and overall PFS (16.3 months) in the gefitinib plus chemotherapy group were numerically comparable with the PFS of osimertinib as first-line treatment in patients with brain metastases (15.2 months).^25^ Thus, the combination treatments of first-generation EGFR-TKIs and chemotherapy is still an attractive option to improve outcomes, especially for patients in good condition.

In the NEJ009 study, individual OS data on a subgroup with brain metastases were not reported, and the OS benefit with gefitinib plus chemotherapy was not significant, with a limited sample size.^17^ In our study, the median OS and 3-year OS rates were significantly longer in the gefitinib plus chemotherapy group than in the gefitinib group, supporting the finding that gefitinib plus chemotherapy was a promising optional first-line treatment in Chinese patients with *EGFR* mutation and brain metastases.

For the mechanism of acquired resistance to EGFR-TKI combination therapy, the NEJ026 study reported that erlotinib combined with bevacizumab had a similar percentage of *EGFR* T790M mutation with erlotinib alone at progression.^27^ In our study, the *EGFR* T790M mutation rate was numerically lower in the gefitinib plus chemotherapy group than in the gefitinib group, although with no statistically significant difference due to the limited sample size. In addition, the PFS on subsequent treatment with third-generation TKIs was not statistically significantly different between the 2 groups. Therefore, the effect of EGFR-TKI combination therapy on the subsequent treatment needs further investigation in a larger sample size.

Radiotherapy has been the traditional method for localized management of brain metastases. The optimal sequence or combination of BRT and targeted therapy in *EGFR*-mutant NSCLC with brain metastases remains controversial. A multicenter retrospective study reported that up-front stereotactic radiosurgery followed by EGFR-TKI treatment had the longest OS compared with whole-brain radiotherapy followed by EGFR-TKIs, and up-front EGFR-TKI treatment.^28^ However, other studies found whole-brain radiotherapy combined with EGFR-TKIs did not prolong the OS more than EGFR-TKIs alone in patients with *EGFR*-mutation with brain metastases.^29,30^ In the present study, we found patients with larger intracranial lesions benefited less from gefitinib plus chemotherapy than those with a better prognosis. The results were consistent with those noted in a practice setting presented in a retrospective study,^28^ which tended to administer up-front BRT to patients with larger intracranial lesions. In our study, considering the further-line treatment upon progression, patients who received both BRT and third-generation TKIs had the longest OS, followed by those who received third-generation TKIs only and those who received BRT only. Although there was a limited sample size, the results of our study suggest that administration of next-generation central nervous system–penetrant TKIs as subsequent treatment is associated with longer survival and may contribute more to OS than BRT in *EGFR-*mutant NSCLC with brain metastases. Previous studies also reported that receiving third-generation *EGFR*-TKIs in all treatment courses is associated with longer survival in advanced *EGFR*-mutated NSCLC.^17,31^

The genomic context of the driver *EGFR* mutation plays a role in target therapy resistance and prior studies analyzing the impact of comutations have identified worse outcomes associated with alterations in other genes, the most important of which is *TP53*,^32,33,34,35,36^ and thus provided the rationale for investigating treatment intensification with chemotherapy in patients with *TP53* mutation. Zhao et al^12^ reported that *EGFR*-mutant NSCLC with concomitant *TP53* mutation favored gefitinib plus apatinib (an oral vascular *EGFR*-2 TKI) than gefitinib plus placebo. In our study, subgroup analysis of patients with next-generation sequencing data demonstrated that those with concomitant *TP53* mutation benefit more from gefitinib combination therapy. Although the limited sample size of subgroup analysis for both trials necessitates cautious interpretation of the findings and restricts their generalization, the promising efficacy of combination therapy in *EGFR*-mutant NSCLC with concomitant *TP53* mutation warrants further verification in prospective randomized clinical trials.

### Limitations

This study has several limitations. First, the study was not blinded and an independent radiology review committee was not established, which may lead to bias. Second, the subsequent treatment after disease progression was not uniform, and treatment was at the discretion of the physician according to progressive patterns, patients’ symptoms, and ECOG status.

## Conclusions

In this randomized clinical trial, gefitinib plus chemotherapy significantly improved intracranial PFS, PFS, and OS compared with gefitinib alone in asymptomatic patients with untreated *EGFR*-mutant NSCLC brain metastases, with manageable adverse events. Combination gefitinib and chemotherapy could be an optional first-line treatment for this patient population.

## Notes

Supplement 1.
Trial Protocol

Supplement 2. **eTable 1.** Disease Progressive Patterns **eTable 2.** Adverse Events in the Intention-to-Treat Population **eTable 3.** Summary of Drug-Related Adverse Events in Intention-to-Treat Population **eTable 4.** Summary of Postprogression Treatments **eFigure 1.** Flowchart of the Trial **eFigure 2.** Subgroup Analyses for Progression-Free Survival **eFigure 3.** Best Percentage Change From Baseline in Target Lesion Size in the Intention-to-Treat Population **eFigure 4.** Percentage of *EGFR* Thr790Met Mutation After First-Line Treatment Progression in the Gefitinib Plus Chemotherapy Group and Gefitinib Group **eFigure 5.** Kaplan-Meier Curves for PFS of Subsequent Third-Generation TKIs in Gefitinib Plus Chemotherapy Group and Gefitinib Group **eFigure 6.** Kaplan-Meier Curves for Overall Survival According to Third-Generation TKIs and Brain Radiotherapy in All Treatment Courses **eFigure 7.** Kaplan-Meier Curves for Overall Survival Incorporating the Subsequent Third-Generation TKIs and Brain Radiotherapy Upon Progression

Supplement 3.
Data Sharing Statement
