# Supplementary material for: Gefitinib Plus Chemotherapy vs Gefitinib Alone in Untreated EGFR-Mutant Non–Small Cell Lung Cancer in Patients With Brain Metastases: The GAP BRAIN Open-Label, Randomized, Multicenter, Phase 3 Study
Source: JAMA Netw Open. 2023 Feb 8;6(2):e2255050. doi: 10.1001/jamanetworkopen.2022.55050 (PMC9909498; doi:10.1001/jamanetworkopen.2022.55050)
Supplement: Supplement 1. — Trial Protocol [file jamanetwopen-e2255050-s001.pdf]

Gefitinib plus chemotherapy versus gefitinib alone in untreated EGFR-mutant non-small cell lung cancer patients with brain metastases (GAP BRAIN): an open-label, randomized, multicenter, phase 3 study

Study Protocol

Principal Investigator: Dr. Likun Chen

Sponsor: Sun Yat-sen University Cancer Center

January, 2014, ver. 1.0

June, 2014, ver. 2.0

October, 2015, ver. 3.0

Protocol Summary

**Official title:**	Gefitinib plus chemotherapy versus gefitinib alone in untreated EGFR-mutant non-small cell lung cancer patients with brain metastases (GAP BRAIN): an open-label, randomized, multicenter, phase 3 study
**Study purpose**	Comparison the intracranial progression-free survival (iPFS) of gefitinib plus chemotherapy versus gefitinib alone in untreated EGFR-mutant non-small cell lung cancer patients with brain metastases.
**Sponsor**	Sun yat-sen university cancer center
	Sun Yat-Sen University Cancer Center<br>Foshan First People's Hospital<br>Meizhou People's Hospital<br>Southern Hospital of Southern Medical University<br>Southern Theater Air Force Hospital<br>Dongguan People's Hospital
**Study design**	A multicenter, phase 3, open-label, randomized controlled trial
**Study subject**	Treatment naive patients with EGFR-mutated non-small cell lung cancer and brain metastases
**Sample size**	160 participants，allocated in 1:1 ratio to gefitinib plus chemotherapy group and gefitinib alone group
**Inclusion criteria**	1) Paitents who was histologically or cytologically confirmed non-small cell lung cancer with EGFR sensitive mutation (exon 19 deletion or 21 L858R mutation);<br>2) Patients who confirmed as having brain metastases by enhanced brain magnetic resonance imaging (MRI); asymptomatic brain metastases or stable brain metastases after mannitol or corticosteroid treatment;<br>3) Patients who had never received therapy (including

	chemotherapy, WBRT, EGFR-TKI and EGFR monoclonal antibody) after diagnosed brain metastases;<br>4) Patients had at least three metastatic lesions in brain, or patients with 1-2 intracranial lesions who were not suitable for brain radiotherapy, or patients with 1-2 intracranial lesions who refused brain radiotherapy, at least one intracranial lesion with the longest diameter of >5 mm;<br>5) Adult patients (≥ 18 years and ≤75 years);<br>6) ECOG Performance Status 0 or 1;<br>7) Life expectancy of at least 12 weeks;<br>8) Haemoglobin⩾10.0 g/dl, Absolute neutrophil count (ANC) ⩾1.5 x 109/L, platelets⩾100 x 109/L. Total bilirubin＜1.5 x upper limit of normal (ULN). ALT and AST < 2.5 x ULN in the absence of liver metastases, or < 5 x ULN in case of liver metastases. Creatinine clearance ^{3} 60ml/min (calculated according to Cockcroft-gault formula).<br>9) Women of childbearing age must take a pregnancy test (serum or urine) within 7 days before enrollment, and the result is negative, and are willing to use appropriate methods of contraception during the test and within 8 weeks after the last administration of the test drug. For males, consent to use an appropriate method of contraception or be surgically sterilized during the trial and within 8 weeks after the last dose of the trial drug;<br>10) Ability to comply with research and follow-up procedures;<br>11) The patient understands and voluntarily signs the written informed consent.
**Exclusion criteria**	1) Patients diagnosed with stage IV or recurrent non-squamous non-small cell lung cancer with brain metastases receiving any

	systemic chemotherapy, immunotherapy or biological therapy (eg targeted therapy such as erlotinib or gefitinib). And folic acid, vitamin B12, and dexamethasone could not be taken as required by the trial protocol.<br>2) The patient has symptoms of intracranial hypertension that cannot be relieved by dehydration treatment, such as nausea or vomiting (except due to factors such as chemotherapy or cerebral infarction); headache; limb movement disorders; epilepsy; cognitive or emotional disorders.<br>3) Concomitant use of phenytoin, carbamazepine, rifampicin, barbital or St. John's wort.<br>4) Previous interstitial lung disease, drug-induced interstitial disease, radiation pneumonitis requiring hormone therapy, or any active interstitial lung disease with clinical evidence, and idiopathic pulmonary fibrosis was found on CT scan at baseline; no Controlled massive pleural or pericardial effusion;<br>5) Any unstable systemic disease (including active infection, poorly controlled hypertension, unstable angina, congestive heart failure, liver, kidney or metabolic disease);<br>6) Any obvious ocular abnormalities, especially severe dry eye syndrome, dry keratoconjunctivitis, severe exposure keratitis, or other diseases that may increase epithelial damage.<br>7) Patients who cannot accept oral administration, require intravenous high-energy nutrition, have previously undergone surgery that affects absorption, or have active peptic ulcers;<br>8) Pregnant or lactating women;<br>9) At the same time, other anti-tumor treatments other than this study were performed.<br>10) Previous radiotherapy to less than 25% of the bone marrow

	was permitted; however, previous radiotherapy to the whole pelvis was not permitted. Prior radiation therapy must have been completed at least 2 weeks prior to the first study drug treatment. Patients must recover from acute toxic effects before first study drug treatment.<br>11) Malignancy other than NSCLC within 5 years prior to randomization, except for adequately treated cervical carcinoma in situ, basal cell or squamous cell skin cancer, localized prostate cancer after radical resection, ductal in situ after radical resection cancer.
**Dosing schedule**	Arm A: gefitinib alone (250mg once daily);<br>Arm B: gefitinib (250mg once daily) plus pemetrexed-platinum chemotherapy (pemetrexed 500mg/m2 combined with cisplatin 75mg/m2 or nedaplatin 80mg/m2) in a 4-week cycle, for four to six cycles, followed by gefitinib plus pemetrexed maintenance. Patients continued treatment until intracranial progressive diseases, unacceptable adverse, or any cause of death.
**Efficacy assessment**	Efficacy assessment is conducted every 8 weeks according to RECIST 1.1 criteria;<br>1. Primary endpoint:<br>Intracranial progression-free survival (iPFS);<br>2. Secondary endpoints<br>(1). Progression-free survival (PFS);<br>(2). Overall survival (OS);<br>(3). Intracranial objective response rate (iORR);<br>(4). Objective response rate (ORR).
**Safety assessment**	All adverse events during study period are monitored, including clinical symptoms, vital signs and laboratory examination abnormalities, with clinical manifestation,

	severity, onset time, duration, management and outcome recorded, and its correlation with study drugs is identified. Assessments of safety are made according to NCI-CTCAE 4.0.
**Statistical methods**	For primary endpoint (iPFS), Kaplan-Meier method was used to estimate the median value and its 95% CI, and the survival graph was depicted. Log-Rank was used to test the survival difference between the two groups. Cox regression models will be applied to estimate differences between treatment groups adjusted for selected prognostic factors (such as sex, age, smoking, mutation type, presence or absence of central nervous system symptoms).<br>For secondary efficacy endpoints, PFS,and OS are generated by Kaplan-Meier method with estimation for median and 95% CI. Survival differences are compared between groups with stratified log-rank test.<br>The comparison of intracranial ORR,total ORR between two group are evaluated using Fisher's exact test.<br>The incidence of adverse events between two groups are compared using Fisher's exact test.
**Study agenda**	Estimated entry time of the first participant: Jan, 2016<br>Estimated entry time of the last participant: Dec, 2020<br>Estimated completion time of the study: Dec, 2021

CONTENTS

1. Background 1
2. Study Purpose 5
    2.1 Study objectives 5
    2.2 Primary endpoint 5
    2.3 Secondary endpoints 6
3. Study Design 7
    3.1 Study design 7
    3.2 Randomization 7
    3.3 Study schema 7
    3.4 Study population 7
    3.5 Inclusion criteria 8
    3.6 Exclusion criteria 8
    3.7 baseline examination 10
    3.8 Treatment period examination 10
    3.9 Primary endpoint 10
    3.10 Secondary endpoints 11
    3.11 Sample Size 11
    3.12 Statistical analysis 12
4. Study Treatment 13
    4.1 Study treatment 13
        4.1.1 Experimental group 13
        4.1.2 Control group 13
    4.2 Dosing schedule 13
    4.3 Dose administration 13
    4.4 Pemetrexed-Related Toxicity, Dose Adjustment or Delay in Treatment 14
        4.4.1 Hematological toxicity 14
        4.4.2 Nephrotoxicity 15
        4.4.3 Grade 3/4 nausea, vomiting 16
        4.4.4 Ototoxicity 16
        4.4.5 Neurotoxicity 16
        4.4.6 Other non-hematologic toxicities 17
        4.4.7 Clinically significant exudation 17
        4.4.8 Insufficient folic acid or vitamin B12 supplementation 18
    4.5 Dose Adjustment or Delay in Patients Receiving Gefitinib 18
    4.6 Blinding 18
    4.7 Concomitant therapy 19
        4.7.1 Colony stimulating factor 19
        4.7.2 NSAIDs 19
        4.7.3 Calcium folinate rescue therapy 19
        4.7.4 Treatment of diarrhea 20
        4.7.5 Treatment of Febrile Neutropenia 20
        4.7.6 Concomitant drugs and gefitinib 20

4.8 Treatment compliance 21
4.9 Efficacy assessment 21
4.9.1 Primary efficacy endpoint 21
4.9.2 Secondary efficacy endpoints..22
4.10 Safety Assessment..23
4.10.1 Safety assessment..23
4.10.2 Adverse Events..23
4.11 Serious Adverse Events..28
4.11.1 Suspected Unexpected Serious Adverse Reactions...29
4.11.2 Other security indicators 29
4.11.3 Drug Safety Monitoring 30
4.12 Ethical Principles and Informed Consent...30
4.12.1 Declaration 30
4.12.2 Ethics...30
4.12.3 Raw data verification 31
4.12.4 Quality Control and Assurance 31
4.12.5 Informed Consent/Data Protection Agreement 31
4.12.6 Modification of clinical protocol..32
4.12.7 Case report form..32
4.12.8 Subject Privacy..32
4.12.9 Publishing papers 32
4.12.10 Data archiving 33
4.13 Study Flowchart..34
Reference 37

1. Background

Lung cancer is one of the most common malignant tumors, and brain metastasis is an important cause of treatment failure and death in patients with lung cancer [1]. The prognosis of brain metastases from lung cancer is poor, and the treatment effect is unsatisfactory, whether it is single or multiple [2]. The incidence of brain metastases in non-small cell lung cancer (NSCLC) is about 30-50%, of which about 25-30% of newly diagnosed NSCLC patients have brain metastases [3]. Studies have shown that patients with lung adenocarcinoma and patients with EGFR gene mutations are prone to brain metastases [4]. In recent years, with the increase in the incidence of lung cancer, the improvement of diagnostic technology and the treatment efficacy of tumors, the overall survival of patients has been prolonged, and the incidence of NSCLC brain metastases has been increasing.

Despite advances in the treatment of NSCLC, brain metastases are still an important factor leading to short survival and poor quality of life. There is no effective treatment for NSCLC brain metastases, and the prognosis is poor [5]. The median survival time of untreated patients is less than 3 months, and the 1-year natural survival rate is less than 10%. Whole brain radiotherapy (WBRT) dominates the treatment of brain metastases, and WBRT can prolong the median survival time of patients to 3-6 months [6, 7]. Whole brain radiotherapy 30Gy/10 times is currently the generally accepted radiotherapy regimen. Single brain metastases can be treated with surgery or stereotactic radiation therapy (SRS). However, most patients with brain metastases from lung cancer cannot receive surgery and SRS because of multiple intracranial lesions, and WBRT has dose limitations, so systemic therapy can be used as a means to control intracranial and extracranial lesions. In the past 10 years, with the emergence of new drugs for the treatment of lung cancer, including pemetrexed, paclitaxel, docetaxel, vinorelbine, gemcitabine, etc., these drugs combined with platinum-based regimens have improved the efficacy of non-small cell lung cancer to more than 40%[8]. The survival time was prolonged, at the same time, the treatment efficacy of brain metastases was improved, and the effective rate of cisplatin-based combination regimen

in the treatment of NSCLC brain metastases was 16%-50%. Brain metastases partially destroy the blood-brain barrier. The median survival (MST) of patients with brain metastases who receive chemotherapy alone is about 4-10 months, and chemotherapy combined with MST is about 6-10 months [8-10]. Therefore, there is still a lack of effective treatments for patients with brain metastases from NSCLC. The emergence of targeted drugs has become a new option in addition to traditional treatment methods such as radiotherapy and chemotherapy.

In recent years, the emergence of epidermal growth factor receptor (EGFR) tyrosine kinase inhibitor (EGFR-TKI) has brought the treatment of non-small cell lung cancer to a new level. Targeted therapy can make advanced non-small cell lung cancer from chemotherapy alone the median survival of 8-10 months improved to nearly 2 years in sensitive patients, represented by gefitinib and erlotinib[11, 12]. EGFR-TKI efficacy is correlated with EGFR mutation status. The EGFR mutation rate in NSCLC is 10% in the Caucasian population and 30-40% in the Asian population. EGFR mutations frequently occur in women, adenocarcinoma, non-smoking, and Asian populations. After first-line treatment with gefitinib in patients with EGFR mutated NSCLC, the objective response rate is 55%-82%, and the median progression-free survival is 8.9-13.3 months [12]. In recent years, large-scale phase III clinical trials have confirmed that gefitinib can be used as a first-line choice for EGFR-mutated non-small cell lung cancer.

The development of EGFR-TKI has brought new options for the treatment of patients with brain metastases from lung cancer, but most of them are retrospective studies and phase II single-arm clinical studies. Several studies reported gefitinib in the treatment of brain metastases from lung cancer, and the efficacy of intracranial lesions was similar to that of extracranial lesions, with an objective response rate of 43%-82.4%, and an overall disease control rate of 45%-100%. A retrospective study showed that in 14 patients with brain metastases from NSCLC treated with gefitinib, the intracranial RR was 43% (CR: 1/14, PR5/14), the extracranial RR was 50%, and the mOS was 9.1 month [13]. Porta R et al reported that 17 patients with EGFR-mutated NSCLC with brain metastases received erlotinib treatment, the objective response rate was 82.4%,

the disease control rate was 100%, PFS and OS were 11.7 months and 12.9 months, respectively [14]. A prospective phase II study by Luchi et al showed that in 21 patients with brain metastases from NSCLC treated with gefitinib (11 first-line and 10 second-line), intracranial RR was 50% and DCR was 90.5%, mPFS was 5 months, mOS was 9.9 months [15]. CTONE-0803 enrolled 48 patients with brain metastases with EGFR mutation or adenocarcinoma. The overall population PFS was 9.7 months, iPFS was 10.1 months, OS was 18.9 months, and the overall response rate was 58.3%. Among them, the number of EGFR mutation cases was 8 (16.7%), and the PFS of the mutated population was up to 15.2 months [16]. Therefore, EGFR-TKI has good efficacy in NSCLC patients with brain metastases, and the 2013 NCCN guidelines pointed out that erlotinib has a certain efficacy in the treatment of brain metastases.

Pemetrexed/platinum is the first-line chemotherapy regimen for non-squamous non-small cell lung cancer. Studies have shown that pemetrexed-based regimens have better efficacy in brain metastases. Ortuzar et al. retrospectively analyzed two large phase III clinical studies and found that treatment with pemetrexed in first- or second-line therapy reduced the risk of brain metastases being the first site of progression. Brain metastases occurred in 3.2% of patients who received pemetrexed and 6.6% of patients who did not receive pemetrexed. This study suggests that pemetrexed has a good efficacy on the prevention of brain metastases. Bearz et al. reported that 39 patients were enrolled in the clinical trial and received pemetrexed as second-line or above treatment, and the overall lesion control rate and brain lesion control rate were 69%. Surprisingly, the control rate of brain lesions was 69%. The rate reached 82%, only 7 patients had brain lesions progression, and the OS was 10 months [17]. Barlesi et al. reported that 43 non-small cell lung cancer patients with brain metastases were treated with pemetrexed/cisplatin as first-line treatment. The response rates of intracranial and extracranial lesions were 41.9% and 34.9%, respectively, and the median survival time was 7.4 months. The PFS was 4.0 months [18]. In general, the pemetrexed/platinum regimen for the patients with non-squamous non-small cell lung cancer with brain metastases is well tolerated, and has a higher effective rate compared with other third-generation chemotherapeutic drugs.

The newly reported FAST-ACT II clinical study [19] showed that compared with gemcitabine/cisplatin chemotherapy alone, gemcitabine/cisplatin combined with erlotinib can improve progression-free survival in the overall population, from 7.4 months to 10 months. Among them, the PFS of the EGFR mutated subgroup increased from 6.9 months to 16.8 months, and the OS increased from 20.6 months to 31.4 months. The proportion of the second-line erlotinib treatment in the chemotherapy group alone was 77% in the overall population. The crossover rate for the mutant subgroup was 83%. Preclinical studies have demonstrated that the combination therapy of erlotinib and pemetrexed has a strong synergistic effect in 6 non-small cell lung cancer cell lines with different molecular properties, supporting the above results [20, 21]. Therefore, we hypothesized that in patients with EGFR-mutated NSCLC and brain metastases, gefitinib combined with pemetrexed/platinum chemotherapy could improve PFS and OS compared with gefitinib alone. We performed this clinical trial to investigate the efficacy and safety of gefitinib plus pemetrexed/platinum chemotherapy in untreated EGFR mutated NSCLC with brain metastases.

2. Study Purpose

2.1 Study objectives

The incidence of brain metastases in non-small cell lung cancer is 30% to 50%. Whole brain radiotherapy combined with chemotherapy is the conventional treatment for non-small cell lung cancer. As a small molecule targeted drug, EGFR-TKI has good cell penetration and can partially penetrate the blood-brain barrier. The 2013 NCCN guidelines pointed out that erlotinib has a certain efficacy on the treatment of brain metastases, and EGFR-TKI has become new options beyond traditional chemotherapy in patients with brain metastases. Therefore, in recent years, researchers are constantly looking for the optimal treatment regimes for non-small cell lung cancer with brain metastases. The CTONE-0803 study showed that second-line EGFR-TKI treatment in patients with EGFR-mutated NSCLC and brain metastases resulted in a PFS of 15.2 months. The OS was 18.9 months, and the overall response rate was 58.3%. In the FAST-ACT2 study, chemotherapy (gemcitabine/carboplatin) combined with EGFR-TKI significantly prolonged PFS and OS in the general population and in patients with EGFR-mutated NSCLC. Retrospective studies suggest that pemetrexed can reduce the incidence of brain metastases in non-small cell lung cancer. Phase II clinical studies have shown that the efficacy of pemetrexed combined with cisplatin or carboplatin can reach 41.9% for intracranial lesions in non-small cell lung cancer. Therefore, we hypothesis that EGFR-TKI combined with pemetrexed/platinum chemotherapy may have better efficacy in untreated non-small cell lung cancer patients with EGFR mutation and brain metastases. There is no study reported the combination of EGFR TKI and chemotherapy in EGFR mutated non-small cell lung cancer. We performed this study to observe and evaluate the efficacy and safety of gefitinib plus chemotherapy in untreated non-small cell lung cancer patients with EGFR mutation and brain metastases.

2.2 Primary endpoint

Comparison of intracranial progression-free survival (iPFS) in gefitinib plus

chemotherapy group and gefitinib alone group.

2.3 Secondary endpoints

(1). Comparison of progression-free survival (PFS) in gefitinib plus chemotherapy group and gefitinib alone group.

(2). Comparison of overall survival (OS) in gefitinib plus chemotherapy group and gefitinib alone group.

(3). Comparison of intracranial objective response rate (iORR) in gefitinib plus chemotherapy group and gefitinib alone group.

(4). Comparison of objective response rate (ORR) in gefitinib plus chemotherapy group and gefitinib alone group.

(5). Comparison of safety in gefitinib plus chemotherapy group and gefitinib alone group.

3. Study Design

3.1 Study design

This study is a randomized, open-label, controlled, multicenter Phase III study. Arm A: gefitinib alone (250mg once daily); Arm B: gefitinib (250mg once daily) plus pemetrexed-platinum chemotherapy (pemetrexed 500mg/m2 combined with cisplatin 75mg/m2 or nedaplatin 80mg/m2) in a 4-week cycle, for four to six cycles, followed by gefitinib plus pemetrexed maintenance. Patients continued treatment until intracranial progressive diseases, unacceptable adverse, or any cause of death.

3.2 Randomization

The eligible patients were randomly assigned (1:1) by a computer-generated randomization sequence to receive gefitinib alone or gefitinib plus pemetrexed-platinum chemotherapy.

3.3 Study schema

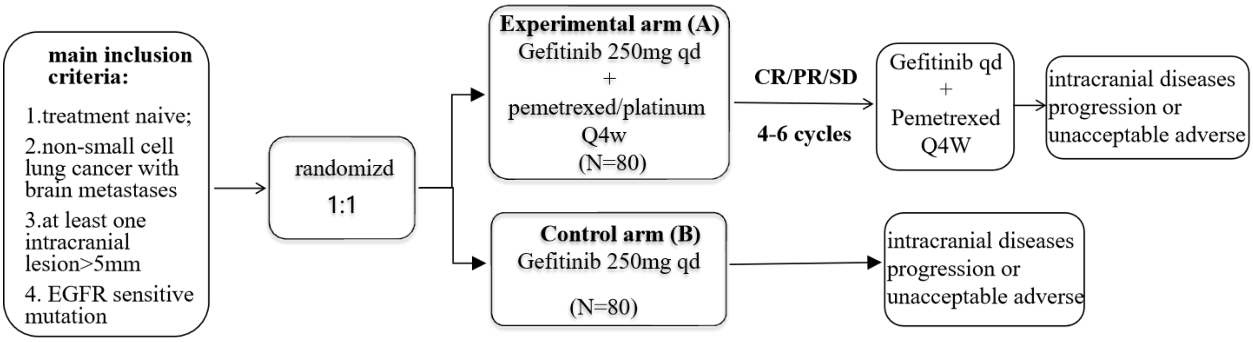

3.4 Study population

This study is conducted in treatment-naïve patients with confirmed NSCLC harboring EGFR mutation and brain metastases. Asymptomatic brain metastases or stable brain metastases after mannitol or corticosteroid treatment.

3.5 Inclusion criteria

1) Paitents who was histologically or cytologically confirmed non-small cell lung cancer with EGFR sensitive mutation (exon 19 deletion or 21 L858R mutation);

2) Patients who confirmed as having brain metastases by enhanced brain magnetic resonance imaging (MRI); asymptomatic brain metastases or stable brain metastases after mannitol or corticosteroid treatment;

3) Patients who had never received therapy (including chemotherapy, WBRT, EGFR-TKI and EGFR monoclonal antibody) after diagnosed brain metastases;

4) Patients had at least three metastatic lesions in brain, or patients with 1-2 intracranial lesions who were not suitable for brain radiotherapy, or patients with 1-2 intracranial lesions who refused brain radiotherapy, at least one intracranial lesion with the longest diameter of >5 mm;

5) Adult patients (≥ 18 years and ≤75 years);

6) ECOG Performance Status 0 or 1;

7) Life expectancy of at least 12 weeks;

8) Haemoglobin⩾10.0 g/dl, Absolute neutrophil count (ANC)⩾1.5 x 109/L, platelets ⩾100 x 109/L. Total bilirubin＜1.5 x upper limit of normal (ULN). ALT and AST < 2.5 x ULN in the absence of liver metastases, or < 5 x ULN in case of liver metastases. Creatinine clearance ³ 60ml/min (calculated according to Cockcroft-gault formula).

9) Women of childbearing age must take a pregnancy test (serum or urine) within 7 days before enrollment, and the result is negative, and are willing to use appropriate methods of contraception during the test and within 8 weeks after the last administration of the test drug. For males, consent to use an appropriate method of contraception or be surgically sterilized during the trial and within 8 weeks after the last dose of the trial drug;

10) Ability to comply with research and follow-up procedures;

11) The patient understands and voluntarily signs the written informed consent.

3.6 Exclusion criteria

Anyone who met any of the following exclusion criteria was not eligible for this trial:

1) Patients diagnosed with stage IV or recurrent non-squamous non-small cell lung cancer with brain metastases receiving any systemic chemotherapy, immunotherapy or biological therapy (eg targeted therapy such as erlotinib or gefitinib). And folic acid, vitamin B12, and dexamethasone could not be taken as required by the trial protocol.

2) The patient has symptoms of intracranial hypertension that cannot be relieved by dehydration treatment, such as nausea or vomiting (except due to factors such as chemotherapy or cerebral infarction); headache; limb movement disorders; epilepsy; cognitive or emotional disorders.

3) Concomitant use of phenytoin, carbamazepine, rifampicin, barbital or St. John's wort.

4) Previous interstitial lung disease, drug-induced interstitial disease, radiation pneumonitis requiring hormone therapy, or any active interstitial lung disease with clinical evidence, and idiopathic pulmonary fibrosis was found on CT scan at baseline; no Controlled massive pleural or pericardial effusion;

5) Any unstable systemic disease (including active infection, poorly controlled hypertension, unstable angina, congestive heart failure, liver, kidney or metabolic disease);

6) Any obvious ocular abnormalities, especially severe dry eye syndrome, dry keratoconjunctivitis, severe exposure keratitis, or other diseases that may increase epithelial damage.

7) Patients who cannot accept oral administration, require intravenous high-energy nutrition, have previously undergone surgery that affects absorption, or have active peptic ulcers;

8) Pregnant or lactating women;

9) At the same time, other anti-tumor treatments other than this study were performed.

10) Previous radiotherapy to less than 25% of the bone marrow was permitted; however, previous radiotherapy to the whole pelvis was not permitted. Prior radiation therapy must have been completed at least 2 weeks prior to the first study drug treatment. Patients must recover from acute toxic effects before first study drug treatment.

11) Malignancy other than NSCLC within 5 years prior to randomization, except for adequately treated cervical carcinoma in situ, basal cell or squamous cell skin cancer,

localized prostate cancer after radical resection, ductal in situ after radical resection cancer.

3.7 baseline examination

1) Pathological examination (histological or cytological examination) and EGFR gene detection;

2) Medical history and physical examination, within 1 week before treatment;

3) ECOG score, within 1 week before treatment;

4) Blood biochemistry examination: including ALT, AST, bilirubin, creatinine, alkaline phosphatase, blood glucose, blood electrolytes, within 1 week before treatment;

5) Blood test: including hemoglobin, red blood cell, leukocyte, neutrophil count, lymphocyte count and platelet count, within 1 week before treatment;

6) ECG examination, within 2 weeks before treatment;

7) Chest CT (unenhanced scan + enhanced), within 3 weeks before treatment;

8) Brain imaging examination, using MRI (unenhanced scan + enhanced), within 3 weeks before treatment.

3.8 Treatment period examination

1) Evaluation of adverse events (AEs): Evaluations were performed every 8 weeks until tumor progression or discontinuation of gefitinib for 1 month. Thereafter, for all AEs that occurred, unless the investigator determined that remission was unlikely due to the patient's underlying disease, all AEs that persisted must be followed until resolution.

2) Evaluation of curative effect: The evaluation method of brain is MRI, and the evaluation method of primary tumor is CT, and the evaluation is carried out every 8 weeks. Evaluation of other extracranial tumors depends on the specific situation of different patients, and the time and method are determined by the investigator.

3) Survival follow-up: every 4 months until the patient died.

3.9 Primary endpoint

Comparison of intracranial progression-free survival (iPFS: defined as time from

randomization to intracranial progressive disease or death.) in gefitinib plus chemotherapy group and gefitinib alone group.

3.10 Secondary endpoints

(1). Comparison of progression-free survival (PFS: time from randomization to overall disease progression or death) in gefitinib plus chemotherapy group and gefitinib alone group.

(2). Comparison of overall survival (OS: time from randomization to death from any cause) in gefitinib plus chemotherapy group and gefitinib alone group.

(3). Comparison of intracranial objective response rate (iORR: proportion of patients with complete or partial response of intracranial lesions) in gefitinib plus chemotherapy group and gefitinib alone group.

(4). Comparison of objective response rate (ORR: proportion of patients with complete or partial response of overall lesions) in gefitinib plus chemotherapy group and gefitinib alone group.

(5). Comparison of safety in gefitinib plus chemotherapy group and gefitinib alone group. Adverse events were graded according to the 4.0th edition of CTCAE of the National Cancer Institute (NCI).

3.11 Sample Size

The sample size calculation of this study is based on the assumption that, for patients with EGFR-mutant NSCLC with brain metastases, gefitinib combined with pemetrexed/platinum therapy can prolong the intracranial disease progression-free time of patients compared with gefitinib alone. Based on iPFS reported in the literature, we determined that gefitinib monotherapy provided an iPFS of 8 months, the HR of gefitinib plus pemetrexed/platinum was 0.67, and the mean iPFS was 12 months. The analysis had 80% power to detect differences at the one-sided 10% significance level. With 80 patients in each group, a cumulative enrollment period of 12 months, and a follow-up period of 12 months. Based on these assumptions, calculations were performed using the PASS 11 software.

3.12 Statistical analysis

The two-sided alpha value was 0.05 for all examinations performed, unless otherwise stated. All interaction checks that will be performed will have a two-sided alpha value of 0.1, and all CIs refer to the two-sided 95% value unless otherwise stated.

The primary endpoint was iPFS. The primary analysis compared iPFS and PFS in the groups receiving gefitinib in combination with pemetrexed/platinum and those receiving gefitinib monotherapy, with a one-sided significance level of 0.10. Cox regression models will be applied to estimate differences between treatment groups adjusted for selected prognostic factors (for stratification, age, smoking history, mutation type, presence or absence of symptoms of intracranial hypertension).

The overall response rate (ORR), defined as the proportion of tumor-response eligible patients with a PR or CR tumor response, will be calculated for each treatment group. The disease control rate (DCR), which is the proportion of randomized patients whose tumor response was PR or CR or SD, will be calculated for each treatment group. ORR and DCR were also calculated as the proportion of patients in the tumor-eligible population undergoing sensitivity analysis. Fisher's exact test will be used for comparison of treatment groups.

The safety analysis of this study will include the total number and incidence (per treatment group) of laboratory and non-laboratory highest-grade CTCAE (version 4) adverse events, and their association with study drug. Toxicity rates will be compared between treatment groups.

4. Study Treatment

4.1 Study treatment

4.1.1 Experimental group

Patients received gefitinib (250mg once daily) plus pemetrexed-platinum chemotherapy (pemetrexed 500mg/m^2 combined with cisplatin 75mg/m^2 or nedaplatin 80mg/m^2) in a 4-week cycle, for four to six cycles, followed by gefitinib plus pemetrexed maintenance. Patients continued treatment until intracranial progressive diseases, unacceptable adverse, or any cause of death.

4.1.2 Control group

Patients received gefitinib alone (250mg once daily) until intracranial progressive diseases, unacceptable adverse, or any cause of death.

4.2 Dosing schedule

Based on clinical data, pemetrexed 500 mg/m^2 was chosen to be administered on the first day of each cycle as an intravenous infusion over 10 minutes, every 28 days as a cycle.

Based on clinical data, cisplatin 25 mg/m^2 intravenous infusion daily on days 1 to 3 of each cycle, or nedaplatin 80 mg/m^2 was administered intravenously on the first day of each cycle, every 28 days as a cycle.

The dose of gefitinib is 250 mg orally daily in 28-day cycles.

4.3 Dose administration

A cycle is defined as an interval of 28 days (period delays due to holidays, weekends and bad weather and other unforeseen circumstances are permissible and not considered against the plan). Each cycle consisted of a combination of pemetrexed and platinum on the first day of treatment. Actual pemetrexed, cisplatin /nedaplatin doses should be calculated based on body surface area before the start of each cycle. A ± 5% difference is allowed between each administered dose and the calculated total dose.

The patients are required oral gefitinib 250 mg once daily. Tablets are taken on an empty

stomach 1 hour before or 2 hours after a meal with 200 ml of water.

4.4 Pemetrexed-Related Toxicity, Dose Adjustment or Delay in Treatment

At the start of subsequent cycles, dose adjustments were made based on the hematologic counts or the maximal non-hematologic toxicity from the previous treatment cycle. Treatment can be delayed to ensure adequate recovery time. Any patients requiring dose reduction continued to receive the reduced dose during the study. Any patient who undergoes two steps of dose reductions and develops toxicity requiring the third dose reduction must be withdrawn from pemetrexed/ platinum therapy. If patients dropped out of pemetrexed/ platinum therapy, they could continue to receive gefitinib rather than withdraw from the study. Treatment can be delayed up to 42 days from Day 1 of the current cycle to allow sufficient time for patients to recover from study drug-related toxicity. Patients who were unable to receive study drug within 42 days of their last treatment had to be withdrawn from pemetrexed/ platinum unless approved by the sponsor to continue the trial.

4.4.1 Hematological toxicity

At the start of subsequent cycles, dose adjustments were made based on the minimum of platelet counts and neutrophil counts from the previous treatment cycle. The absolute neutrophil count (ANC) must be ⩾1.5 x 109/L and the platelet count must be ⩾100 x 109/L prior to the start of any cycle. Blood tests should be performed for each patient within 7 days prior to randomization, within 4 days prior to dosing of each cycle (except for cycle 1, which can be performed within 7 days prior to randomization), and at the first follow-up visit after the end of treatment. school inspection. Treatment can be delayed to allow sufficient time for the patient to recover. Recovered patients must be re-treated according to the guidelines provided in Table 4.1. Hematological toxicities not listed in Table 4.1 do not require dose adjustment.

Table 4.1 Dose adjustment for pemetrexed /nedaplatin based on hematologic toxicity from previous cycle

Platelet (x10 9 /L) minimum value	ANC (x10 9 /L) minimum value	pemetrexed	nedaplatin

≥ 50	and	≥ 0.5	100%	100%
≥ 50	and	< 0.5	75%	75%
< 50	and	any	75%	75%
< 50 with bleeding	and	any	50%	50%
any	and	<1.0+ fever ≥38.5 ℃	75%	75%
3 or 4 grade Thrombocytopenia after two steps of reduction	or	3 or 4 grade neutropenia after two steps of reduction	Patient withdrawal	Patient withdrawal

4.4.2 Nephrotoxicity

(1) Dose adjustment of cisplatin

Muscular hepatic clearance (Ccr) before treatment should be ≥ 60 ml/min, and should be used before the start of each treatment cycle.

Cockcroft-Gault formula for evaluation.

$$\text{Male: } \frac{[140 - \text{age}] \times \text{actual weight (kg)}}{72 \times \text{serum creatinine (mg/dL)}} = \text{mL/min}$$

Female: male creatinine clearance rate × 0.85

For chemotherapy after cycles 2 and 2, if Ccr < 60 ml/min, check serum creatinine after 24 hours of hydration and calculate creatinine clearance using the Cockcroft-Gault formula, dose adjustment of cisplatin is performed according to the following criteria . If 24-hour urine collection is used to estimate Ccr, the result calculated by the above formula is no longer used, and measures are taken according to the following method:

(If the results calculated by the formula differ significantly from those estimated from collected urine, consider using the most accurate result).

Table 4.2 Dose adjustment for cisplatin based on Nephrotoxicity toxicity from previous cycle

≥60ml/min	100% of the original dose;
41-59 ml/min	Numerical dose of cisplatin (mg/m ^{2}) and Ccr (ml/min) are the same value (for example: if the Ccr is 45 ml/min, the dose of cisplatin is 45mg/m^2);
≤40ml/min	Permanently discontinue cisplatin.

(2) Dosage adjustment plan for pemetrexed

Pemetrexed is mainly excreted in the original form through the urinary tract. No dose adjustment of pemetrexed is required if the patient's creatinine clearance is ≥45 mL/min. For patients with creatinine clearance <45 mL/min, there are insufficient patient data to give a recommended dose. Therefore, pemetrexed should not be given to patients with creatinine clearance <45 mL/min calculated according to the Cockcroft-Gault formula.

(3) Dose adjustment scheme of nedaplatin

No dose adjustment is required for nedaplatin if the patient's creatinine clearance is ≥45 mL/min . Nedaplatin should not be administered to patients with creatinine clearance <45 mL/min calculated according to the Cockcroft-Gault formula .

The interval for retesting of creatinine clearance is recommended weekly but must be based on the opinion of the investigator. If the patient's creatinine clearance does not return to the above level (CrCl>45 mL/min) within 42 days after the previous pemetrexed dose , the patient must discontinue chemotherapy .

4.4.3 Grade 3/4 nausea, vomiting

If the patient develops grade 3/4 nausea or vomiting, the dose of cisplatin should be reduced to 60 mg/m 2 in subsequent courses , or the dose of nedaplatin should be reduced to 75% of the original dose .

4.4.4 Ototoxicity

If the patient develops hearing loss, new tinnitus, or new severe high-frequency hearing on the hearing curve

If strength is lost, cisplatin /nedaplatin should be discontinued . However, if there is clinical benefit, patients can continue to be treated with pemetrexed and gefitinib.、

4.4.5 Neurotoxicity

When neurotoxicity occurred, dose adjustments for pemetrexed/ platinum and gefitinib are shown in Table 4.5 . Cisplatin should be discontinued if grade 3 or 4 neurotoxicity occurs, but pemetrexed can be continued in patients with clinical benefit.

Table 4.3 : Dose Adjustments for Pemetrexed / Cisplatin/Nedaplatin and Gefitinib Due to Neurotoxicity

CTC grading	Pemetrexed / nedaplatin/ gefitinib dose	Cisplatin dose
0-1	100 % of original dose	100 % of original dose
2	100 % of original dose	50 % of the original dose

4.4.6 Other non-hematologic toxicities

In general, in the event of a nonhematologic toxicity greater than or equal to the Common Terminology Criteria for Adverse Events (CTCAE) grade 3, treatment must be delayed until less than or equal to the patient's baseline value, after which treatment can be resumed. At the start of subsequent cycles, dose reductions were made based on nonhematologic toxicities that occurred after treatment in the previous cycle.

Table 4.3. Dose Adjustment of Pemetrexed Based on Nonhematologic Toxicity in the Previous Cycle

CTCAE grade	% of previous dose
3rd or 4th grade nausea or vomiting	100%
2 grade transaminases	100%
3 grade transaminases	100%
4 grade transaminases	75%
3rd or 4th grade mucositis	50%
3rd or 4th grade diarrheaa	75%
Grade 0 to 2 neurotoxicity	100%
Grade 3 or 4 neurotoxicityb	Discontinuation of pemetrexed therapy
Other 3rd or 4th grade non-hematologic CTCAE	75%
Grade 3 or 4 CTCAE recurrence after 2 dose reductions	Discontinuation of pemetrexed therapy

4.4.7 Clinically significant exudation

If patients had clinically significant pleural effusion or ascites prior to randomization, biologics could be given to control pleural effusion, but pleural infusion of chemotherapy drugs was not allowed. and through drainage or other surgical treatment uncontrolled, patients should be excluded from the study.

If a patient develops clinically significant pleural effusion or ascites (as determined by symptoms or clinical examination) during treatment, drainage of the effusion should be considered prior to administration. However, patients should be discontinued from the study if the investigator believes that the effusion is indicative of disease progression.

4.4.8 Insufficient folic acid or vitamin B12 supplementation

Folic acid should be taken for at least 3 days and an injection of vitamin B12 should be given within 7 days before the first dose of pemetrexed. When pemetrexed is given later, folic acid should be taken for at least 14 days in the 28 days before Day 1 of the next cycle before pemetrexed is given.

4.5 Dose Adjustment or Delay in Patients Receiving Gefitinib

If the patient develops intolerable diarrhea (sometimes associated with dehydration), adverse cutaneous drug reactions, or any adverse event deemed by the investigator to be attributable to gefitinib treatment, short-term (up to 14 days) interruption and then reinstating the patient on 250 mg daily may successfully treat these adverse events.

In the event of an acute onset or exacerbation of pulmonary symptoms (dyspnea, cough, fever), gefitinib treatment should be discontinued and these symptoms should be investigated immediately and appropriate treatment initiated. If interstitial lung disease is diagnosed, gefitinib should be discontinued and the patient given appropriate treatment.

Patients with new-onset ocular symptoms, such as pain, should be medically evaluated and given appropriate treatment, including discontinuation of gefitinib and removal of trichiasis if they develop. After symptoms and ocular changes resolve, then physician decide whether to resume 250 mg daily therapy.

No dose adjustment is required when patients differ in age, weight, sex, race, or renal status, or in the presence of moderate to severe hepatic impairment due to liver metastases.

Patients in group A (gefitinib combined with chemotherapy) can continue to receive chemotherapy if gefitinib therapy is discontinued.

4.6 Blinding

This is an open label clinical trial.

4.7 Concomitant therapy

Patients were allowed to receive comprehensive supportive care concurrently during the study. No other chemotherapy, immunotherapy, cancer stimulation veterinary therapy, surgery, or other investigational drugs. Pain that is not effectively controlled by systemic therapy or local analgesia of metastatic lesions, allowing the use of palliative radiotherapy to irradiate small areas. If any other specific anticancer therapy is required due to disease progression, study treatment must be discontinued early. Particular attention should be paid to the following concomitant treatments:

4.7.1 Colony stimulating factor

The use and prevent use of granulocyte colony-stimulating factor (G-CSF), erythropoietin (EPO), and thrombopoietin-stimulating drugs (TPO) in accordance with CSCO's standardized management guidelines for tumor radiotherapy and chemotherapy-related myelosuppression are allowed.

4.7.2 NSAIDs

Patients taking NSAIDs or salicylates (excluding low-dose aspirin [≤1.3 g/day]), NSAIDs should be discontinued 2 days before, on the day of, and 2 days after receiving pemetrexed. If patients are taking long-half-life NSAIDs (eg, naproxen, piroxicam, diflunisal, or nerbulimone), they should be discontinued 5 days before, on the day of, and 2 days after receiving pemetrexed. The use of opioid analgesics is not limited.

4.7.3 Calcium folinate rescue therapy

Since folic acid and vitamin B12 supplementation significantly reduced the number of episodes of grade 4 hematologic and grade 3/4 nonhematologic toxicities associated with pemetrexed treatment, the use of calcium folinate as a rescue agent is not expected. However, this section provides information for when rescue medication is needed. In clinical trials, leucovorin is permitted for CTCAE grade 4 leukopenia lasting >3 days, CTCAE Grade 4 neutropenia lasting >3 days and should be used immediately when CTCAE Grade 4 thrombocytopenia, Grade 3 thrombocytopenia related bleeding, or Grade 3 or 4 mucositis occurs. When calcium folinate is administered intravenously,

the following usage and dosage are recommended: 100 mg/m^2 for the first time, and 50 mg/m^2 every 6 hours thereafter for 8 days.

4.7.4 Treatment of diarrhea

Patients with diarrhea should be given standard antidiarrheal therapy according to local routine practice. Patients with CTCAE grade 3 or 4 diarrhea should be given intravenous fluids, octreotide, and antidiarrheals. If diarrhea is severe (requiring intravenous fluids) or associated with fever or grade 3 or 4 neutropenia, broad-spectrum antibiotics should be given. Patients with severe diarrhea and severe nausea or vomiting must be hospitalized, given intravenous fluids, and corrected for electrolyte imbalances.

4.7.5 Treatment of Febrile Neutropenia

Patients with febrile neutropenia with or without diarrhea should be seen in the hospital according to standard management procedures, and intravenous antimicrobial therapy should be initiated urgently.

4.7.6 Concomitant drugs and gefitinib

Gefitinib may interact with CYP3A4 inhibitors and CYP3A4 promoters; therefore, the study protocol does not allow the use of CYP3A4 inducers or inhibitors in therapy. Drugs that induce CYP3A4, such as rifampicin or phenytoin, can reduce the efficacy of gefitinib by increasing its metabolism and reducing its plasma concentration. Conversely, CYP3A4 inhibitors, such as ketoconazole and itraconazole, can increase the efficacy of gefitinib by decreasing its metabolism and increasing its plasma concentration.

Elevated INR and/or increased INR have been reported in patients taking warfarin concomitantly with gefitinib bleeding events. If the patient requires anticoagulation, it is recommended to use low molecular weight heparins (if clinically available) as opposed to coumarins. However, if coumarins are required after randomization , patients should be regularly monitored for changes in PT or INR.

4.8 Treatment compliance

pemetrexed/ platinum is administered, it should only be administered intravenously at the study center. This ensures that the patient's obedience. Patients randomized to receive pemetrexed/ platinum should be supplemented with vitamin B12 administered intramuscularly at the study center. This ensures patient compliance. Adherence to folic acid and dexamethasone supplementation needs to be monitored through patient interviews.

With regard to gefitinib, patient compliance with study medication will be assessed at each visit. By comparison retrieved and dispensed tablets to assess adherence. Substantially poor compliance has been demonstrated (one visit for patients taking <80% or >120% of the intended study drug at intervals, the study center must inform the patient of the importance of study drug adherence and drug counts. Patients who continue to fall outside compliance will be discontinued treatment.

4.9 Efficacy assessment

The purpose of this study was to evaluate the safety and efficacy of gefitinib plus pemetrexed/platinum chemotherapy versus gefitinib monotherapy. The primary efficacy measure was the comparison of intracranial progression-free survival (iPFS) between the two groups.

Secondary efficacy measures included a comparison of progression-free survival (PFS) between gefitinib plus pemetrexed/platinum chemotherapy versus gefitinib monotherapy for non-small cell lung cancer with brain metastases; The time of symptom control of intracranial hypertension in each treatment mode; the remission rate of brain metastases in the two treatment modes; the cognitive function of the two groups of patients; the 24-month and 36-month survival rates and overall survival (OS) of the two group.

4.9.1 Primary efficacy endpoint

Intracranial Progression-free Survival (iPFS): From the date of randomization to the first observation of intracranial disease progression (based on imaging; or the first

occurrence of intracranial disease in patients with asymptomatic brain metastases before enrollment) One of the symptoms of hypertension [nausea or vomiting (except due to factors such as chemotherapy or cerebral infarction), headache, cognitive or affective disorders, epilepsy, limb movement disorders)]; or patients with symptoms of intracranial hypertension before enrollment, who have been treated effectively Time interval (days) from control to re-increase of intracranial hypertension symptoms; or death from any cause.

Tumor progression was defined as at least a 20% increase in the sum of the LD of the measured lesions or the appearance of one or more new lesions, referenced to the smallest overall longest diameter (LD) value recorded since the start of treatment. The appearance of any new lesions is indicative of disease progression. In exceptional cases, definitive progression of unmeasurable lesions is also accepted as evidence of disease progression. Imaging progression was determined using the judgment of an independent data review committee. Patients who were alive and did not progress by the date of analysis will be cut off on the date of their last imaging assessment.

4.9.2 Secondary efficacy endpoints

Progression-free survival (PFS): from the date of randomization to the first observation of intracranial or extracranial disease progression (intracranial based on imaging or intracranial hypertension, whichever is extracranial imaging) If patients died from other causes before disease progression, the number of days from randomization to death was calculated.

Intracranial Objective Response Rate (iORR): The proportion of patients who achieved a complete or partial response with the best intracranial response .

Overall objective response rate (ORR): The proportion of patients with a complete or partial response to the overall best response.

Overall survival (OS): Time from randomization to death from any cause. Patients still alive at the time of analysis will have the date of their last contact as the cutoff date.

Cognitive function: Hopkins Vocabulary Learning Test-Revised (Hopkins Vethal Leaming Test-Revise, HVLT-R) and Trail Marking Test (TMT) were used to evaluate.

4.10 Safety Assessment

All patients who received at least one study drug treatment will be included as the safety population for safety analysis. The patient's physical examination, vital signs, adverse events, and laboratory abnormalities were summarized. all adverse events should be reported and graded according to the NCI Terminology Criteria for Common Adverse Events (CTCAE) version 4.0.

4.10.1 Safety assessment

(1) Vital signs and physical examination

Patients will undergo a comprehensive physical examination, including ECOG score, physical examination, height, weight, and measurements of vital signs such as heart rate, blood pressure, temperature, and respiratory rate.

(2) Laboratory examination

Blood routine, blood biochemistry, urine routine and screening pregnancy tests will be performed in the clinical laboratories of each research center.

This study will evaluate the following laboratory metrics.	
**Blood chemistry (serum gel tubes)**	
Alanine aminotransferase (ALT)	Urea nitrogen (BUN)
Aspartate aminotransferase (AST)	Creatinine (Cr)
Alkaline Phosphatase (ALP)	Potassium ion (K)
Total Bilirubin (TB)	Sodium ion (Na)
Total protein (TP)	Calcium ions (Ca)
Albumin (ALB)	

**Blood test (ethylenediaminetetraacetic acid [EDTA] tubes)**	
Neutrophil Count (ANC)	White Blood Cell Count (WBC)
Hemoglobin (HGB)	Platelet count (PLT)
Red blood cell count (RBC)	

4.10.2 Adverse Events

Subjects must be closely monitored for adverse events. Such monitoring includes

clinical laboratory examinations. Adverse events should be assessed in terms of severity, severity, and relationship to the test drug.

The investigator is responsible for evaluating the relationship between all adverse events and the study drug. However, the Principal Investigator may entrust the judgment of other qualified clinicians participating in this study, but remains responsible. Investigators must provide a list of qualified and commissioned personnel.

(1) Definition of adverse events

An adverse event is an unforeseen medical condition or deterioration of a pre-existing medical condition that occurs during or after the use of a drug, whether related to the study drug or not. Unforeseen medical conditions may be symptoms (eg, nausea, chest pain), signs (eg, tachycardia, enlarged liver), or abnormal test results (eg, laboratory tests, electrocardiogram). In clinical research, from the signed informed consent form, adverse events can be unforeseen adverse medical conditions that occur at any time, including screening or washout periods, even if have not received study treatment.

Adverse events in humans (whether drug-related or not) include the following:

- Adverse events that occur during the use of the drug by professionals;
- Adverse events resulting from drug overdose (whether intentional or unintentional);
- Adverse events caused by drug abuse;
- Adverse events caused by discontinuation of the drug;
- Adverse events that may arise purely from patient participation in the study (eg, due to discontinuation of antihypertensive drugs during the washout period or serious adverse events) must be reported as adverse events even if not related to the study medication.

Those who did not appear or did not achieve the expected clinical pharmacological effects and have been recorded in the corresponding section of the CRF are not regarded as adverse events. However, if the criteria for "serious" adverse events are met, they should also be recorded and reported as serious adverse events. In this study, any event clearly caused by disease progression was not reported as an adverse event.

(2) Unexpected adverse events

Unexpected adverse events are defined as any characteristic or severity consistent with the Investigator's Brochure (or instructions) inconsistent adverse drug reactions. Characteristics or severity of known, documented adverse events with important information on the extent of the unexpected adverse event is also part of the reporting. For example: (a) acute renal failure that has been identified as an adverse event followed by interstitial nephritis; and (b) first report hepatitis with acute hepatic necrosis.

(3) Observation, recording and reporting of adverse events

All adverse events that occurred after the subject signed the informed consent form must be completely recorded in the subject's case report form.

Documentation must be supported by original sources. Each event should be described in detail, including the date of beginning and discontinuation, severity, relationship to the test product, actions taken, and outcome of the event.

① How to detect adverse events

At each visit, adverse events can be detected by:

- Unsolicited information from patients or caregivers;
- At each visit, ask the patient open, non-leading questions: How are you feeling? Have you had any (other) medical problems since your last visit?
- Abnormalities observed by investigators, other medical staff, and family members

② Time to collect adverse events

In this study, non-serious adverse events were recorded from the time the patient gave informed consent until the patient 30-day follow-up period after withdrawal from treatment.

③ Collection of adverse event data

All adverse events should be recorded on the CRF. The description of the adverse event includes its start and end time, whether it is in line with serious adverse events, measures taken (such as changes in study treatment, other treatments and follow-up examinations) and outcomes, and the investigator is asked to evaluate the causal relationship (with the study treatment). Relationship). Adverse events should be graded

by NCI CTC and their changes recorded on the corresponding CRF.

(4) Criteria for judging the severity of adverse events

The severity of adverse events should be recorded according to the National Cancer Institute-Common Toxicity Criteria Version 4.0 (NCI–CTCAE 4.0).

(5) Judgment criteria for the relationship between adverse events and trial drugs

Clinicians should assess the probability that an AE is related to experimental drug therapy and classify the probability into 5 categories ‘definitely related, probably related, definitely unrelated, probably unrelated, unclassified’. Of which ‘definitely related’, ‘probably related’ and ‘unclassified’ are all seen as AE. The incidence of AE is calculated as the sum of AE cases (‘definitely related’ + ‘probably related’ + ‘to be determined’) divided by number of patients evaluable for safety. The criteria for causality assessment is shown in table 4.4.

Table 4.4. Criteria for assessment of causal relationship between AE and experimental drug

Classification	Criteria for assessment
definitely related	The lag time prior to onset of the event is consistent with a reasonable chronological order after the last dose of the drug, and the event fits the characteristics of known reaction to the drug or other drugs of the same class; the severity of the event has reduced on drug dose interruption, and the manifestations of the event reappear after the drug is restarted.
probably related	The lag time prior to onset of the event is consistent with a reasonable chronological order after the last dose of the drug, but the event does not fit the characteristics of known reaction to the drug or other drugs of the same class; it seems possible that the underlying clinical condition of the patient or one of the concomitant medications known to cause the event actually has caused it.
probably unrelated	The lag time prior to onset of the event is not consistent with a reasonable chronological order after the last dose of the drug, and the event does not fit the characteristics of known reaction to the drug or other drugs of the same class; it seems possible that the underlying clinical condition of the patient or one of the concomitant medications known to cause the event actually has caused it.
definitely unrelated	The lag time prior to onset of the event is not consistent with a reasonable chronological order after the last dose of the drug, and the event does not fit the characteristics of known reaction to the drug or other drugs of the same class; it seems possible that the underlying clinical condition of the patient or one of the concomitant medications known to cause the AE actually has caused it; the manifestations of the event disappear on the disease amelioration or concomitant medications interruption, and reappear after concomitant medications are restarted.
unclassified	The lag time prior to onset of the event is ambiguously consistent with a reasonable chronological order after the last dose of the drug, but the event fits the characteristics of known reaction to the drug or other drugs of the same class; it seems possible that one of the concomitant medications known to cause the event actually has caused it.

Five criteria for adverse reaction/event analysis:

1. Is there a reasonable time relationship between the medication and the occurrence of adverse reactions/events?

2. Does the reaction match the type of adverse reaction known to the drug?

3. Did the reaction disappear or lessen after discontinuation or dose reduction?

4. Does the same reaction/event occur again when the suspected drug is used again?

5. Can the reaction/event be explained by the effect of concomitant medications, patient progression, effects of other treatments?

If it is considered to be definitely related, likely related and possibly related, it should be regarded as an adverse reaction caused by the drug, and it should be considered as a serious adverse event according to the severity.

In this study, the abnormality of simple laboratory test indicators was judged by the investigator whether it had clinical significance, and those without clinical significance were not reported as adverse events. In the event of an unexplained abnormal laboratory test result, the test result should be repeated immediately and followed up until it returns to the normal range and/or is sufficient to explain the abnormality. Record clear explanations on the case report form.

(6) Treatment, follow-up and duration of adverse event cases

Close follow-up of the patient after the investigator's initial report of an adverse event is required to provide further information to the study sponsor. All adverse events during the study, even if the patient has completed the study or ended treatment, should be followed up until remission or until the patient's condition is stable, unless according to the investigator's judgment, the adverse event is unlikely to be alleviated due to the patient's own condition, or patients were lost to follow-up.

4.11 Serious Adverse Events

(SAEs) were collected from the time patients signed informed consent and received study drug . If a patient developed an SAE after giving informed consent but before receiving study drug, this event will not be collected unless the investigator believes that the event was caused by a protocol procedure.

Surgery that was previously planned (before signing the ICF) should not be reported as an SAE unless the underlying disease worsened during the course of the study.

In the event of any serious adverse event (SAE), site personnel must report the event to the study sponsor within 24 hours of becoming aware of the event. Immediately after reporting by telephone, a study-specific SAE form should be completed as a formal notification. This 24-hour notification requirement refers to the initial initial SAE information and all follow-up follow-up SAE information. A SAE is any AE in a study that results in one of the following:

- Death, except due to disease progression (see "Exclusions" below);
- lead to or prolong the hospital stay;

- life-threatening conditions (risk of immediate death);
- Persistent or significant disability/disability;
- Congenital anomalies/birth defects;
- Other reasons judged by the investigator to be serious

Medical events that are important, but do not result in death, life-threatening disease, or require hospitalization, if, using appropriate medical judgment, they are likely to endanger the patient and require medical and surgical intervention to prevent any of the outcomes listed in this definition, then This event can be considered a serious adverse drug event.

Exclusions: Death due to disease progression will only be reported to the study sponsor as an SAE if the investigator believes it is related to the use of the study drug.

Serious adverse events that occurred within 30 days of the patient's last dose were collected, regardless of the investigator's view of causality. Thereafter, reporting of SAEs is not required unless the investigator believes the event is related to the study drug, or the drug delivery system, or the operation of the protocol.

Information on SAEs in the study population that are not expected to be related to drug exposure, and information on SAEs that will be reviewed periodically by the sponsor during the course of the clinical trial. In a population with advanced non-small cell lung cancer, the incidence of serious infections, cardiovascular events, and weight loss can be reasonably predicted based on a variety of factors, such as age, comorbidities, and disease status.

4.11.1 Suspected Unexpected Serious Adverse Reactions

Suspected Unexpected Serious Adverse Reactions (SUSAR) were defined as serious, not listed in the Investigator's Brochure, investigators identify events that are believed to be related to the study drug or procedure.

4.11.2 Other security indicators

Other safety measures include physical examination (to identify rash or third space fluid

accumulation, and possible signs suggestive of AE (such as body temperature, weight, blood pressure, and heart rate) and toxicity grade.

The National Cancer Institute (NCI)-CTCAE v4.0 was used as a reference when selecting appropriate terms and grading severity. When an AE had no matching term in the NCI-CTCAE v 4.0 criteria, the investigator was responsible for selecting the appropriate system organ class and rating the severity of the event based on the intensity of the event. Note that both the CTCAE term (actual or numbered) and severity must be selected by site personnel and recorded on the case report form (CRF). This record is a supplement to the text describing the AE verbatim. Before each cycle, patients will be graded for toxicity (applying the NCI-CTCAE v4.0 criteria).

4.11.3 Drug Safety Monitoring

Throughout the course of the study, the clinical study physician will monitor safety data.

4.12 Ethical Principles and Informed Consent

4.12.1 Declaration

This clinical trial will be conducted in strict compliance with protocol, GCP and regulatory and SOP requirements.

4.12.2 Ethics

It is the investigator's responsibility to provide the ethics committee with the clinical trial protocol, informed consent, and information to obtain an independent approval document for the conduct of the clinical study.

The approval document of the ethics committee must be obtained before the start of the clinical study. Approval from the ethics committee must be in writing and sent to the investigator, who will then provide a copy of the approval to the study sponsor. The ethics committee approval document must be accompanied by a list of all committee members involved in the discussion of the approval document and their respective responsibilities.

During the course of the clinical study, any issues related to the safety of the clinical

study, such as the clinical study protocol or changes to subjects' informed consent and serious adverse events in clinical research must be reported to the ethics committee. The end or early termination of a clinical study must also be reported to the ethics committee.

4.12.3 Raw data verification

For data recorded directly on the case report form (i.e. data not previously recorded in writing or electronically) with and the identification of the raw data considered as the original data should be stipulated and clearly stated in the monitoring plan in advance according to the plan. Otherwise, it is regarded as lack of raw data.

Investigators must properly handle all data obtained during clinical research to ensure the rights and privacy of subjects in clinical trial. The investigator must agree with the monitor/auditor/inspector on the required clinical research data are reviewed and reviewed in order to verify the accuracy of the original data and understand the progress of the research. If the original records cannot be verified, the investigator should agree to assist the monitor/auditor/inspector further confirm the quality of the data.

4.12.4 Quality Control and Assurance

All drugs and materials used in clinical research must be subject to quality control. sponsor, sponsor persons authorized by the party or relevant medical management institutions have the right to conduct audits of clinical research, the purpose is to ensure the authenticity of clinical research record data and comply with the provisions of the clinical research protocol. clinical research subjects will be informed that there will be relevant personnel to audit during the trial, but patient privacy and data will be strictly protected.

4.12.5 Informed Consent/Data Protection Agreement

The investigator is responsible for explaining the purpose, methods, benefits and potential risks of this clinical trial to each subject, and obtaining an informed consent form signed by the subjects of the clinical trial. Before the start of the procedure, the

subject's informed consent must be obtained. For subjects who can not signed the informed consent, the informed consent must be signed by their parents, legal guardians or guardians. By signing an informed consent form, subjects must also consent to the monitor/auditor/health survey organization checks the raw data obtained about the clinical study in order to determine the reliability of clinical study data results.

The original informed consent form signed and dated by the subjects of the clinical trial must be properly kept by the investigator , and the signed informed consent form must also be recorded in the case report form and related original records of the trial.

4.12.6 Modification of clinical protocol

The modification of the clinical protocol requires the joint participation of the sponsor and the investigator. A revised version of the protocol should be submitted to the ethics committee will be approved. Only then can the contents of the revised version be executed.

4.12.7 Case report form

The investigator must ensure that the electronic case report form is completed and accurately completed. Only record in each case report form record data of a clinical study subject.

4.12.8 Subject Privacy

Researchers must ensure that the privacy of clinical trial subjects is maintained. In all documents submitted to the sponsor, the identity of the clinical trial subjects can only be determined by the trial patient number and initials, and cannot be specified subject's full name. Investigators must properly keep the names and addresses of clinical trial subjects and the corresponding entry form. These enrollment forms are kept strictly confidential by the investigator and cannot be submitted to the sponsor.

4.12.9 Publishing papers

For multicenter clinical studies, data from each center cannot be individually published.

The sponsor reserves the right to review the first draft prior to publication or publication of the study results.

4.12.10 Data archiving

The investigators should retain all relevant records for a period of at least 5 years after completion of the study in compliance with Chinese GCP requirements.

4.13 Study Flowchart

	Screening period		Treatment period			Survival follow-up
	-21-0 days	-7-0 days	0 week (+/- 7days)	8 week (+/- 7days)	8n(n=2,3...) (+/- 7 days)	4 months
visit	1		2	3	4,5...	
Informed consent	×					
demography	×					
Medical/surgical history [a]	×					
Smoking history [a]	×					
Previous radiation/chemotherapy	×					
Pregnancy test [b]	×					
Inclusion/Exclusion criteria [c]	×		×			
Physical examination [d]		×	×	×	×	
Intracranial hypertension symptoms		×	×	×	×	
ECOG score		×	×	×	×	
Concomitant medication [e]	×			×	×	
ECG	×			×	×	
Blood test [f]		×		×	×	
Blood biochemistry [f]		×		×	×	
Urine test [f]		×		×	×	
Intracranial tumor assessment [g]	×	×		×	×	
Extracranial tumor assessment [g]	×	×		×	×	
Cognitive function and quality of life assessment [h]		×		×	×	
Toxicity assessment/ adverse events [i]				×	×	
Survival data and anti-tumor therapy [j]						×

Footnotes:

a. A complete medical and surgical history should be obtained including all relevant disorders. In particular, a history of past and current pulmonary disease and/or systemic disease involving the lungs (eg, connective tissue disease) should be asked. Only ask patients about their smoking status at screening.

b. Premenopausal women of childbearing potential must have a negative urine or serum pregnancy test within 7 days before the first dose. If the result is positive, the subject will not be eligible to participate in the study. If pregnancy is suspected during the test, the test should be repeated.
c. If assessments were performed within 7 days before randomization and the listed inclusion and exclusion criteria (if applicable) were met, repeats were not required on the day of the first dose unless the investigator believed that significant changes were likely.
d. Physical examination includes heart rate, blood pressure, respiratory rate, temperature, height, weight, and neurological examination. If body weight was measured at screening, repeating is not necessary unless clinically indicated. Body weight should be measured at screening and when clinically indicated.
e. Data on concomitant medications, including drug dose, route of administration, dosing schedule, start date, indication, end date, and end reason, were collected and must be recorded from randomization with study drug to 1 month after discontinuation. Treating physicians obtained this information from patients' hospital visits every 6 weeks. With knowledge of the patient's routinely prescribed medications, the treating physician will assess whether changes in the patient's concomitant medications over the past 8 weeks are likely to lead to clinically meaningful improvement in pulmonary symptoms.
f. The screening period blood routine/blood biochemistry/urine routine examination should be performed within 7 days before randomization, and the subsequent blood routine, blood biochemistry and urine routine examination should be performed within 7 days before and after the time point specified in the study flow chart. Routine blood tests were performed every 3 days in each phase of chemotherapy, and blood biochemical tests were performed every four weeks and when clinically indicated.
g. Enhanced magnetic resonance imaging for intracranial lesions and enhanced computerized tomography scans for extracranial lesions. Each investigator decides which imaging modality to use, but screening assessments must adequately define aspects of the disease and document measurable lesions according to RECIST 1.1 criteria. Afterwards, repeat imaging studies (every 8 weeks according to the study plan) should also follow RECIST 1.1 guidelines, which require the use of the same assessment methods and the same techniques at screening and follow-up visits for each identifiable and reported lesions. No additional scans are required to confirm CR and PR. If new lesions are suspected at any site, imaging studies should be performed as appropriate. Patients who withdrew from the study not due to disease progression were recommended for objective tumor assessment every 8 weeks to collect information on disease progression.
h. Relevant questionnaires were completed by the researcher through the communication between the researcher and the subjects at each visit. The baseline questionnaire should be completed prior to the first dose. Subsequent questionnaires should be completed on the day of each visit, at the end of study treatment.

i. In the event of any perceived ocular symptoms, new or worsening respiratory symptoms (eg, cough, wheezing), the patient should be given immediate appropriate medical attention. Any symptoms were handled as clinically routine and, if defined, were reported as adverse events or serious adverse events (SAEs). Adverse events were collected from patients signing informed consent. After study termination, all unhealed or serious adverse events were followed until resolution, unless the investigator determined that remission was unlikely due to the patient's own disease.

j. After recording disease progression, investigators were required to contact the patient, the patient's family, or the patient's current treating physician by telephone at least once every 4 months to obtain information on the patient's overall survival and post-study chemotherapy until the patient's death. The following information should be obtained during each follow-up visit: disease status, records of all new anticancer treatments, and date of death (if applicable).

Reference

1. Preusser, M., et al., *Brain metastases: pathobiology and emerging targeted therapies.* Acta Neuropathol, 2012. **123**(2): p. 205-22.
2. Eichler, A.F., et al., *The biology of brain metastases-translation to new therapies.* Nat Rev Clin Oncol, 2011. **8**(6): p. 344-56.
3. Goncalves, P.H., et al., *Risk of brain metastases in patients with nonmetastatic lung cancer: Analysis of the Metropolitan Detroit Surveillance, Epidemiology, and End Results (SEER) data.* Cancer, 2016. **122**(12): p. 1921-7.
4. Rangachari, D., et al., *Brain metastases in patients with EGFR-mutated or ALK-rearranged non-small-cell lung cancers.* Lung Cancer, 2015. **88**(1): p. 108-11.
5. Thomas, S.S. and E.M. Dunbar, *Modern multidisciplinary management of brain metastases.* Curr Oncol Rep, 2010. **12**(1): p. 34-40.
6. Mehta, M.P., et al., *Motexafin gadolinium combined with prompt whole brain radiotherapy prolongs time to neurologic progression in non-small-cell lung cancer patients with brain metastases: results of a phase III trial.* Int J Radiat Oncol Biol Phys, 2009. **73**(4): p. 1069-76.
7. Mehta, M.P., et al., *Survival and neurologic outcomes in a randomized trial of motexafin gadolinium and whole-brain radiation therapy in brain metastases.* J Clin Oncol, 2003. **21**(13): p. 2529-36.
8. Edelman, M.J., et al., *Outcomes associated with brain metastases in a three-arm phase III trial of gemcitabine-containing regimens versus paclitaxel plus carboplatin for advanced non-small cell lung cancer.* J Thorac Oncol, 2010. **5**(1): p. 110-6.
9. Guerrieri, M., et al., *A randomised phase III study of palliative radiation with concomitant carboplatin for brain metastases from non-small cell carcinoma of the lung.* Lung Cancer, 2004. **46**(1): p. 107-11.
10. Antonadou, D., et al., *Phase II randomized trial of temozolomide and concurrent radiotherapy in patients with brain metastases.* J Clin Oncol, 2002. **20**(17): p. 3644-50.
11. Rosell, R., et al., *Erlotinib versus standard chemotherapy as first-line treatment for European patients with advanced EGFR mutation-positive non-small-cell lung cancer (EURTAC): a multicentre, open-label, randomised phase 3 trial.* Lancet Oncol, 2012. **13**(3): p. 239-46.
12. Maemondo, M., et al., *Gefitinib or chemotherapy for non-small-cell lung cancer with mutated EGFR.* N Engl J Med, 2010. **362**(25): p. 2380-8.
13. Park, S.J., et al., *Efficacy of epidermal growth factor receptor tyrosine kinase inhibitors for brain metastasis in non-small cell lung cancer patients harboring either exon 19 or 21 mutation.* Lung Cancer, 2012. **77**(3): p. 556-60.
14. Porta, R., et al., *Brain metastases from lung cancer responding to erlotinib: the importance of EGFR mutation.* Eur Respir J, 2011. **37**(3): p. 624-31.
15. Iuchi, T., et al., *Phase II trial of gefitinib alone without radiation therapy for Japanese patients with brain metastases from EGFR-mutant lung adenocarcinoma.* Lung Cancer, 2013. **82**(2): p. 282-7.
16. Wu, Y.L., et al., *Erlotinib as second-line treatment in patients with advanced non-small-cell lung cancer and asymptomatic brain metastases: a phase II study (CTONG-0803).* Ann Oncol, 2013. **24**(4): p. 993-9.
17. Bearz, A., et al., *Activity of Pemetrexed on brain metastases from Non-Small Cell Lung Cancer.*

Lung Cancer, 2010. **68**(2): p. 264-8.
18. Barlesi, F., et al., *Pemetrexed and cisplatin as first-line chemotherapy for advanced non-small-cell lung cancer (NSCLC) with asymptomatic inoperable brain metastases: a multicenter phase II trial (GFPC 07-01).* Ann Oncol, 2011. **22**(11): p. 2466-2470.
19. Wu, Y.L., et al., *Intercalated combination of chemotherapy and erlotinib for patients with advanced stage non-small-cell lung cancer (FASTACT-2): a randomised, double-blind trial.* Lancet Oncol, 2013. **14**(8): p. 777-86.
20. Li, T., et al., *Schedule-dependent cytotoxic synergism of pemetrexed and erlotinib in human non-small cell lung cancer cells.* Clin Cancer Res, 2007. **13**(11): p. 3413-22.
21. Giovannetti, E., et al., *Molecular mechanisms underlying the synergistic interaction of erlotinib, an epidermal growth factor receptor tyrosine kinase inhibitor, with the multitargeted antifolate pemetrexed in non-small-cell lung cancer cells.* Mol Pharmacol, 2008. **73**(4): p. 1290-300.
