# Supplementary material for: Gefitinib Plus Chemotherapy vs Gefitinib Alone in Untreated EGFR-Mutant Non–Small Cell Lung Cancer in Patients With Brain Metastases: The GAP BRAIN Open-Label, Randomized, Multicenter, Phase 3 Study
Source: JAMA Netw Open. 2023 Feb 8;6(2):e2255050. doi: 10.1001/jamanetworkopen.2022.55050 (PMC9909498; doi:10.1001/jamanetworkopen.2022.55050)
Supplement: Supplement 2. — eTable 1. Disease Progressive Patterns eTable 2. Adverse Events in the Intention-to-Treat Population eTable 3. Summary of Drug-Related Adverse Events in Intention-to-Treat Population eTable 4. Summary of Postprogression Treatments eFigure 1. Flowchart of the Trial eFigure 2. Subgroup Analyses for Progression-Free Survival eFigure 3. Best Percentage Change From Baseline in Target Lesion Size in the Intention-to-Treat Population eFigure 4. Percentage of EGFR Thr790Met Mutation After First-Line Treatment Progression in the Gefitinib Plus Chemotherapy Group and Gefitinib Group eFigure 5. Kaplan-Meier Curves for PFS of Subsequent Third-Generation TKIs in Gefitinib Plus Chemotherapy Group and Gefitinib Group eFigure 6. Kaplan-Meier Curves for Overall Survival According to Third-Generation TKIs and Brain Radiotherapy in All Treatment Courses eFigure 7. Kaplan-Meier Curves for Overall Survival Incorporating the Subsequent Third-Generation TKIs and Brain Radiotherapy Upon Progression [file jamanetwopen-e2255050-s002.pdf]

Supplementary Online Content

Hou X, Li M, Wu G, et al. Gefitinib plus chemotherapy vs gefitinib alone in untreated *EGFR*-mutant non-small cell lung cancer in patients with brain metastases: the GAP BRAIN open-label, randomized, multicenter, phase 3 study. *JAMA Netw Open*. 2023;6(2):e2255050. doi:10.1001/jamanetworkopen.2022.55050

eTable 1. Disease Progressive Patterns

eTable 2. Adverse Events in the Intention-to-Treat Population

eTable 3. Summary of Drug-Related Adverse Events in Intention-to-Treat Population

eTable 4. Summary of Postprogression Treatments

eFigure 1. Flowchart of the Trial

eFigure 2. Subgroup Analyses for Progression-Free Survival

eFigure 3. Best Percentage Change From Baseline in Target Lesion Size in the Intention-to-Treat Population

eFigure 4. Percentage of *EGFR* Thr790Met Mutation After First-Line Treatment Progression in the Gefitinib Plus Chemotherapy Group and Gefitinib Group

eFigure 5. Kaplan-Meier Curves for PFS of Subsequent Third-Generation TKIs in Gefitinib Plus Chemotherapy Group and Gefitinib Group

eFigure 6. Kaplan-Meier Curves for Overall Survival According to Third-Generation TKIs and Brain Radiotherapy in All Treatment Courses

eFigure 7. Kaplan-Meier Curves for Overall Survival Incorporating the Subsequent Third-Generation TKIs and Brain Radiotherapy Upon Progression

This supplementary material has been provided by the authors to give readers additional information about their work.

© 2023 Hou X et al. *JAMA Network Open.*

eTable 1. Disease progressive patterns.

	Patients, No. (%)		
	Gefitinib plus Chemotherapy group	Gefitinib group	*P* value
**Disease progressive pattern,**			.39
Intracranial progression only	23 (37.7)	27 (38.6)	
Extracranial progression only	11 (18.0)	7 (10.0)	
Systemic progression [a]	27 (44.3)	36 (51.4)	
**Intracranial progression,**[b]			.05
Occurring new lesions	4 (8.0)	14 (22.2)	
Existing lesions enlarge	22 (44.0)	17 (27.0)	
Both	24 (48.0)	32 (50.8)	
**Extracranial progression,**[c]			.28
Occurring new lesions	2 (5.3)	0 (0.0)	
Existing lesions enlarge	23 (60.5)	30 (63.8)	
Both	13 (34.2)	17 (36.2)	
**CNS symptom,**[b]			> .99
Symptomatic progression	12 (24.0)	16 (25.4)	
Asymptomatic progression	38 (76.0)	47 (74.6)	

Note: [a] Systemic progression means patients occurred intracranial and extracranial diseases progression simultaneously. [b] Intracranial progression included intracranial progression only and systemic progression. [c] Extracranial progression included extracranial progression only and systemic progression. *P* value was assessed using chi-square test.

© 2023 Hou X et al. *JAMA Network Open.*

eTable 2. Adverse Events in the Intention-to-Treat Population[a]

Adverse events	Patients, No. (%)			
	Gefitinib (n = 81)		Gefitinib plus chemotherapy (n = 80)[b]	
	Any grade	≥3 Grade	Any grade	≥3 Grade
No. of patients	75 (92.6)	17 (21.0)	80 (100.0)	32 (40.0)
Leukopenia	6 (7.4)	0	50 (62.5)	3 (3.8)
Neutropenia	6 (7.4)	0	49 (61.2)	6 (7.5)
Anemia	25 (30.9)	1 (1.2)	45 (56.3)	3 (3.8)
Thrombocytopenia	2 (2.5)	0	19 (23.8)	1 (1.3)
Hyperkalemia	1 (1.2)	0	3 (3.8)	0
Hypokalemia	16 (19.8)	0	11 (13.8)	0
Hyponatremia	13 (16.0)	0	9 (11.3)	0
Hypercalcemia	1 (1.2)	0	5 (6.3)	0
Hypocalcemia	13 (16.0)	0	19 (23.8)	0
Hypoalbuminemia	21 (25.9)	0	30 (37.5)	0
Alanine aminotransferase increase	42 (51.9)	12 (14.8)	56 (70.0)	9 (11.3)
Aspartate aminotransferase increase	41 (50.6)	6 (7.4)	46 (57.5)	2 (2.5)
Alkaline phosphatase increase	31 (38.3)	1 (1.2)	45 (56.3)	0
Blood bilirubin increase	11 (13.6)	1 (1.2)	7 (8.8)	0
Blood creatinine increase	6 (7.4)	1 (1.2)	22 (27.5)	1 (1.3)
Nausea	3 (3.7)	0	40 (50.0)	6 (7.5)
Vomiting	1 (1.2)	0	32 (40.0)	3 (3.8)
Anorexia	15 (18.5)	0	58 (72.5)	4 (5.0)
Constipation	4 (4.9)	0	18 (22.5)	0
Fatigue	20 (24.7)	0	37 (46.3)	2 (2.5)
Rash	42 (51.9)	1 (1.2)	45 (56.3)	2 (2.5)
Pruritus	29 (35.8)	0	26 (32.5)	1 (1.3)
Diarrhea	26 (32.1)	0	20 (25.0)	4 (5.0)
Paronychia	9 (11.1)	0	6 (7.5)	1 (1.3)

[a]All adverse events were evaluated according to National Cancer Institute Common Terminology Criteria for Adverse Events, version 4.0.
[b]Chemotherapy comprised pemetrexed, 500 mg/m^2, combined with cisplatin, 75 mg/m^2, or nedaplatin, 80 mg/m^2, in a 4-week cycle for 4 to 6 cycles, followed by pemetrexed, 500 mg/m^2, as maintenance every 4 weeks.

© 2023 Hou X et al. *JAMA Network Open.*

eTable 3. Summary of drug-related adverse events in intention-to-treat population.

	Patients, No. (%)		
	Gefitinib plus chemotherapy (n=80)	Gefitinib (n=81)	*P* value
Patients with any grade AE	80 (100.0)	75 (92.6)	.03
Patients with grade 3 or worse AE	32 (40.0)	17 (21.0)	.01
Treatment interruption because of AE	10 (12.5)	7 (8.6)	.45
Gefitinib changed to other TKI due to AE	2 (2.5)	6 (7.4)	.28
icotinib	0	4 (4.9)	
afatinib	2 (2.5)	0	
osimertinib	0	2 (2.5)	
Chemotherapy discontinued due to AE	3 (3.8) [a]	NA	NA
Deaths caused by AE	1 (1.3) [b]	0	.50

Note: [a] three patients discontinued chemotherapy due to AEs (one with exfoliative dermatitis; one with crissum abscess; and one with creatinine elevation). [b] one death due to pneumonitis occurred in gefitinib plus chemotherapy group that was considered treatment-related. P value was assessed using Fisher exact test.
Abbreviations: AE, adverse event; TKI, tyrosine kinase inhibitor; NA, not available.

© 2023 Hou X et al. *JAMA Network Open.*

eTable 4. Summary of postprogression treatments.

	Patients, No.	
Variable	Gefitinib plus chemotherapy (n=80)	Gefitinib (n=81)
No. with ongoing first-line treatment	18	10
No. of discontinued first-line treatment	61	70
Lost follow-up during first-line treatment	1	1
No subsequent treatment	6	6
Brain radiotherapy	20	24
WBRT	13	15
SRS	7	9
Any subsequent systemic treatment	55	64
Pemetrexed/platinum with or without bevacizumab	13	28
Paclitaxel or gemcitabine chemotherapy	19	12
Third-generation TKIs	40	46
Other first- or second-generation TKIs	6	5
Anlotinib	5	8
Crizotinib	2	2
Clinical trial	2	4
Other treatment	3	9

Abbreviations: WBRT, whole brain radiation therapy. SRS, stereotactic radiosurgery. TKI, tyrosine kinase inhibitor.

© 2023 Hou X et al. *JAMA Network Open.*

eFigure 1. Flowchart of the trial.

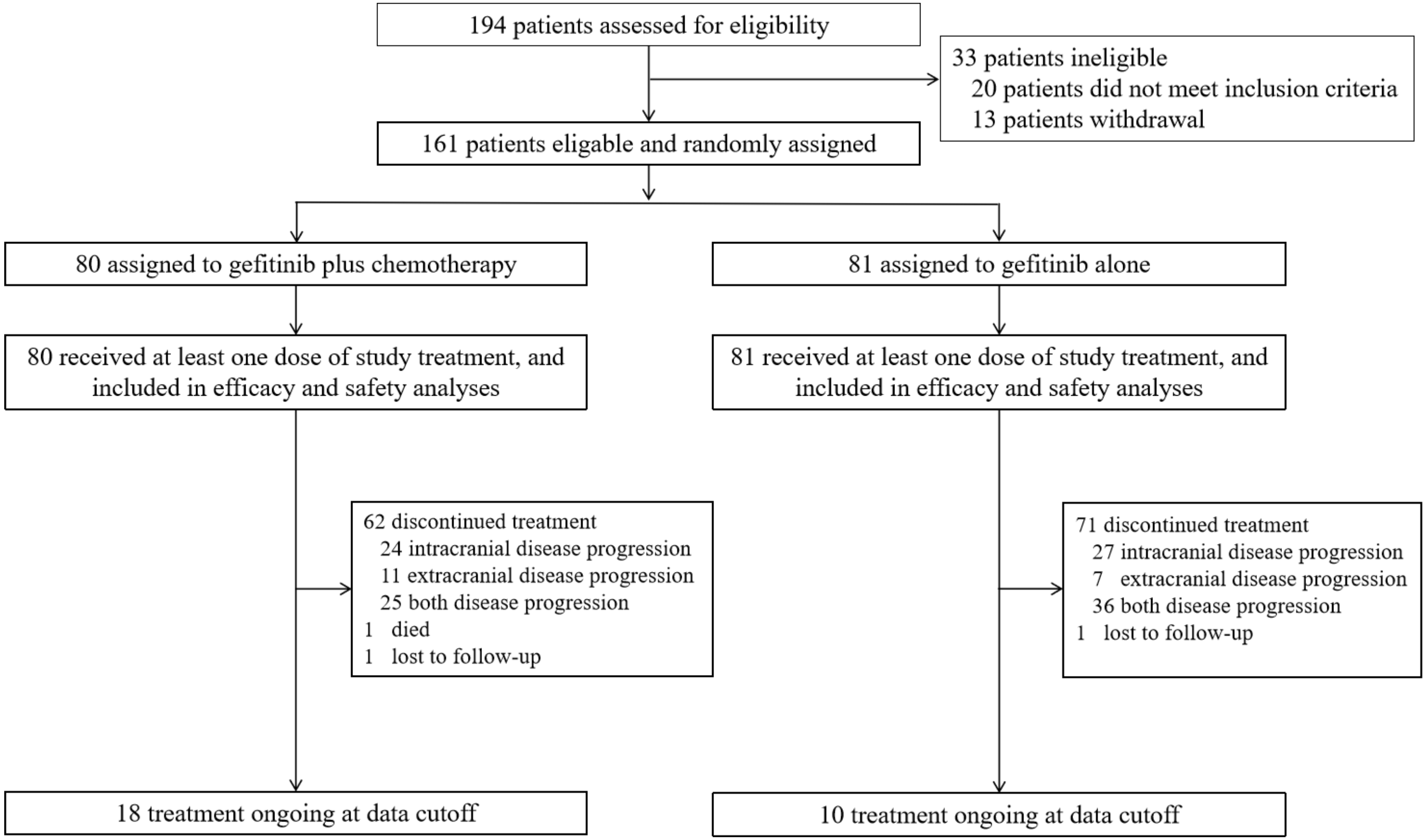

© 2023 Hou X et al. *JAMA Network Open.*

eFigure 2. Subgroup analyses for progression-free survival.

Characteristics	No. of Events/No. of Patients	HR (95%CI)	P Value
Sex			
Female	53/87	0.33 (0.18-0.59)	<0.001
Male	57/74	0.45 (0.26-0.78)	0.004
Age at screening, years			
<65	90/127	0.4 (0.26-0.62)	<0.001
≥65	20/34	0.42 (0.15-1.2)	0.107
Smoking status			
Non-smokers	80/120	0.36 (0.22-0.57)	<0.001
Smokers	30/41	0.43 (0.2-0.91)	0.027
ECOG performance status			
0	27/43	0.31 (0.13-0.73)	0.007
1	83/118	0.42 (0.27-0.66)	<0.001
EGFR mutation type			
Exon 19Del	58/85	0.32 (0.18-0.57)	<0.001
Exon L858R	49/70	0.43 (0.24-0.77)	0.004
TP53 mutation			
Wild-type	14/22	0.37 (0.11-1.28)	0.117
Mutated-type	38/62	0.18 (0.08-0.43)	<0.001
Extracranial metastases			
No	29/43	0.34 (0.15-0.76)	0.009
Yes	81/118	0.39 (0.24-0.62)	<0.001
All patients	110/161	0.39 (0.27-0.58)	<0.001

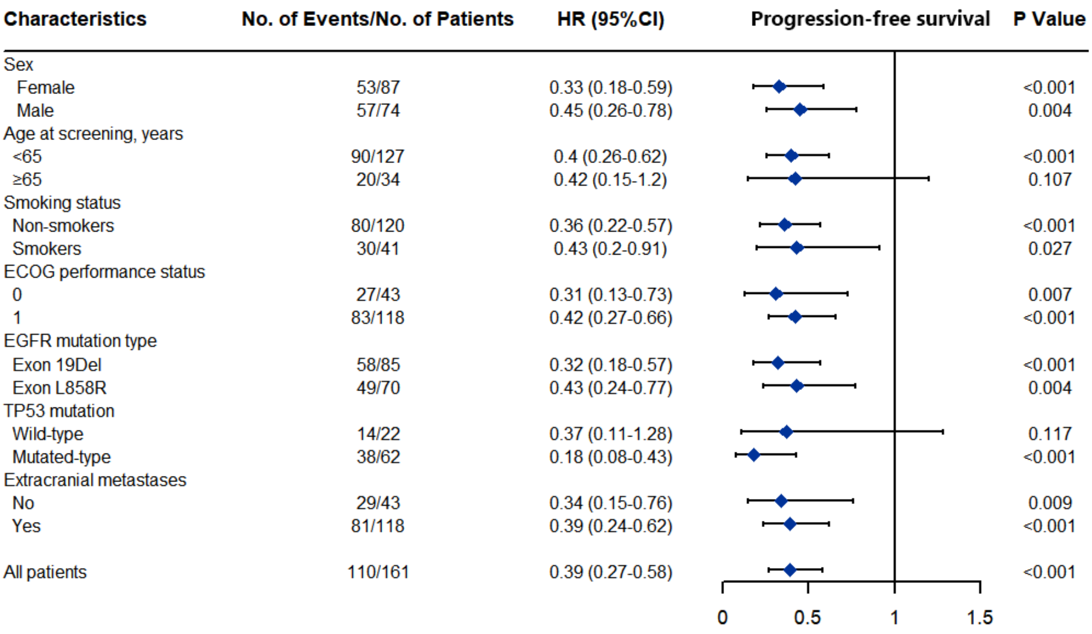

HR and corresponding 95% CI were evaluated using Cox proportional hazards regression model. ECOG, Eastern Cooperative Oncology Group; EGFR, epidermal growth factor receptor; Del, deletion; PFS, progression-free survival; HR, hazard ratio; CI, confidence interval.

© 2023 Hou X et al. *JAMA Network Open.*

eFigure 3. Best percentage change from baseline in target lesion size in the intention-to-treat population. (A) intracranial tumor change; (B) total tumor change.

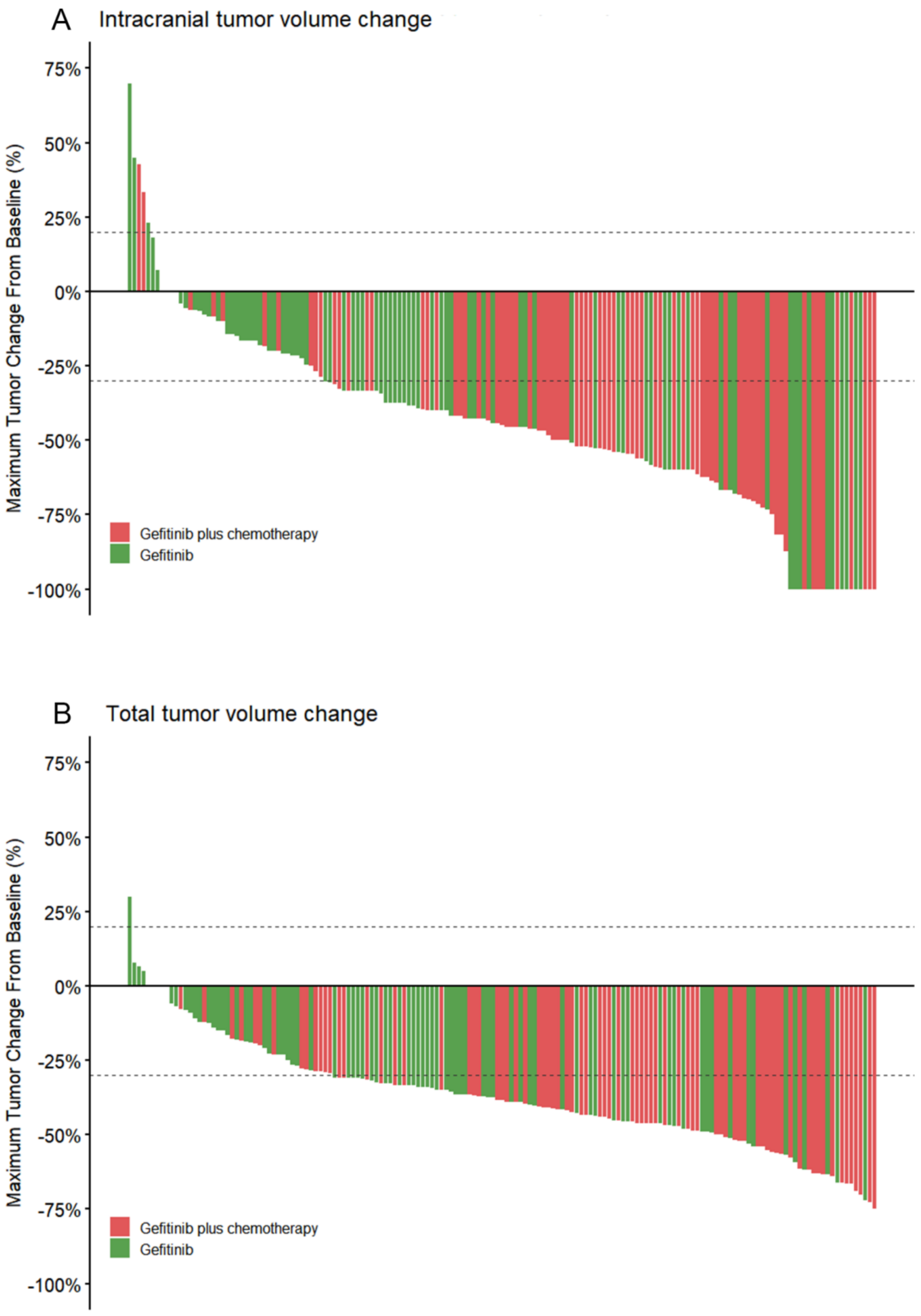

© 2023 Hou X et al. *JAMA Network Open.*

eFigure 4. Percentage of *EGFR* Thr790Met mutation after first-line treatment progression in gefitinib plus chemotherapy group and gefitinib group.

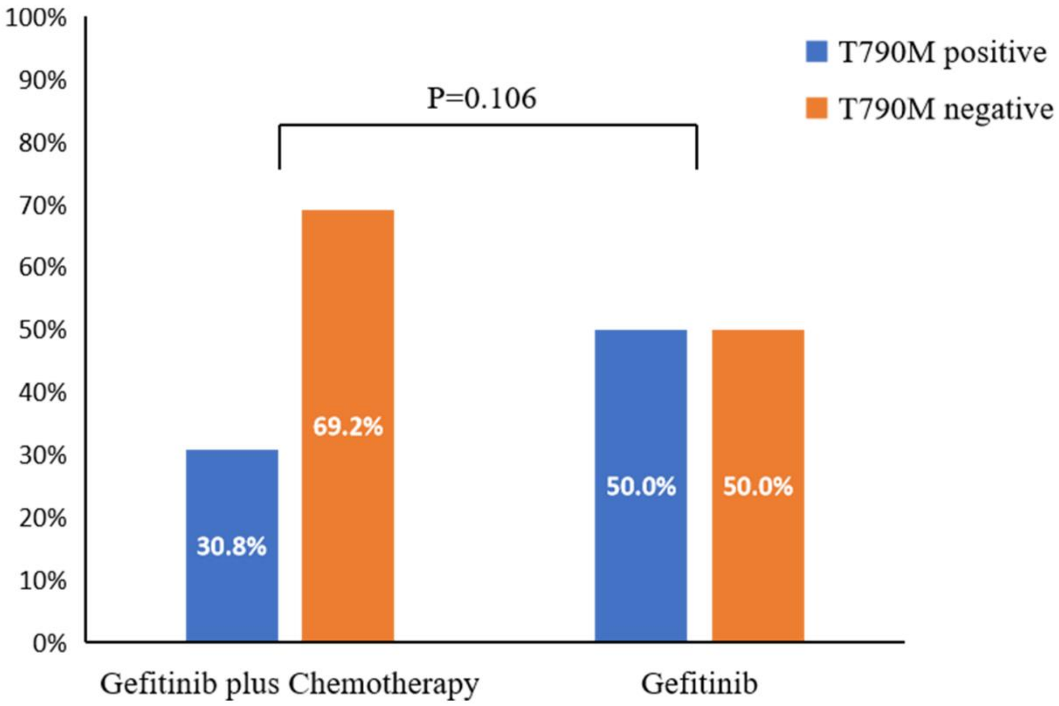

P value was assessed using Fisher exact test.

© 2023 Hou X et al. *JAMA Network Open.*

eFigure 5. Kaplan-Meier curves for PFS of subsequent third-generation TKIs in gefitinib plus chemotherapy group and gefitinib group.

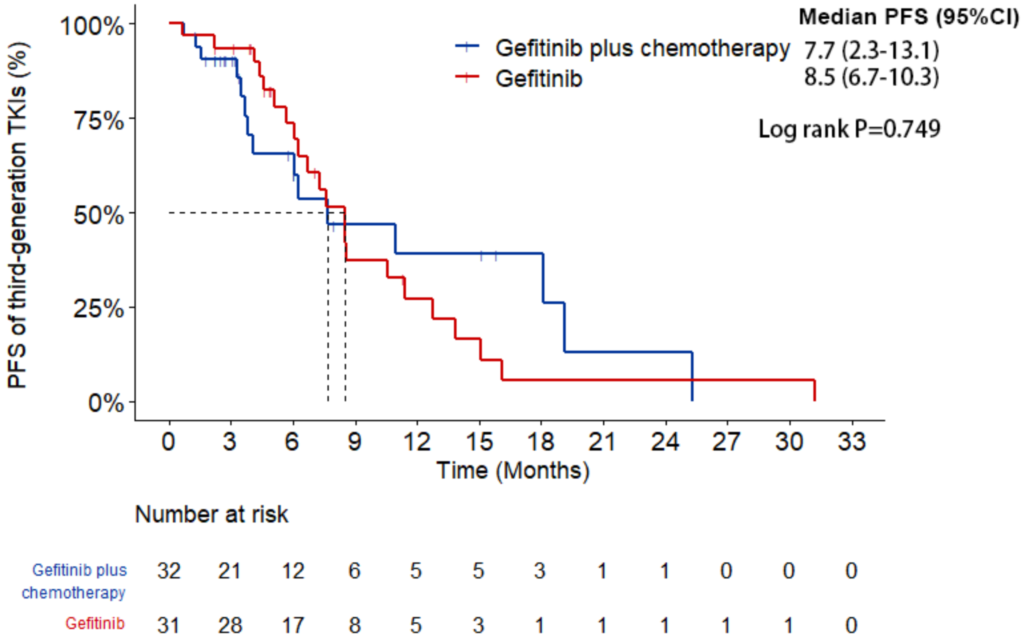

Third-generation TKIs included osimertinib and almonertinib. PFS, progression-free survival; TKIs, tyrosine kinase inhibitors; CI, confidence interval.

© 2023 Hou X et al. *JAMA Network Open.*

eFigure 6. Kaplan-Meier curves for overall survival according to third-generation TKIs and brain radiotherapy in all treatment courses. (A) patients received third-generation TKIs or not; (B) patients received brain radiotherapy or not.

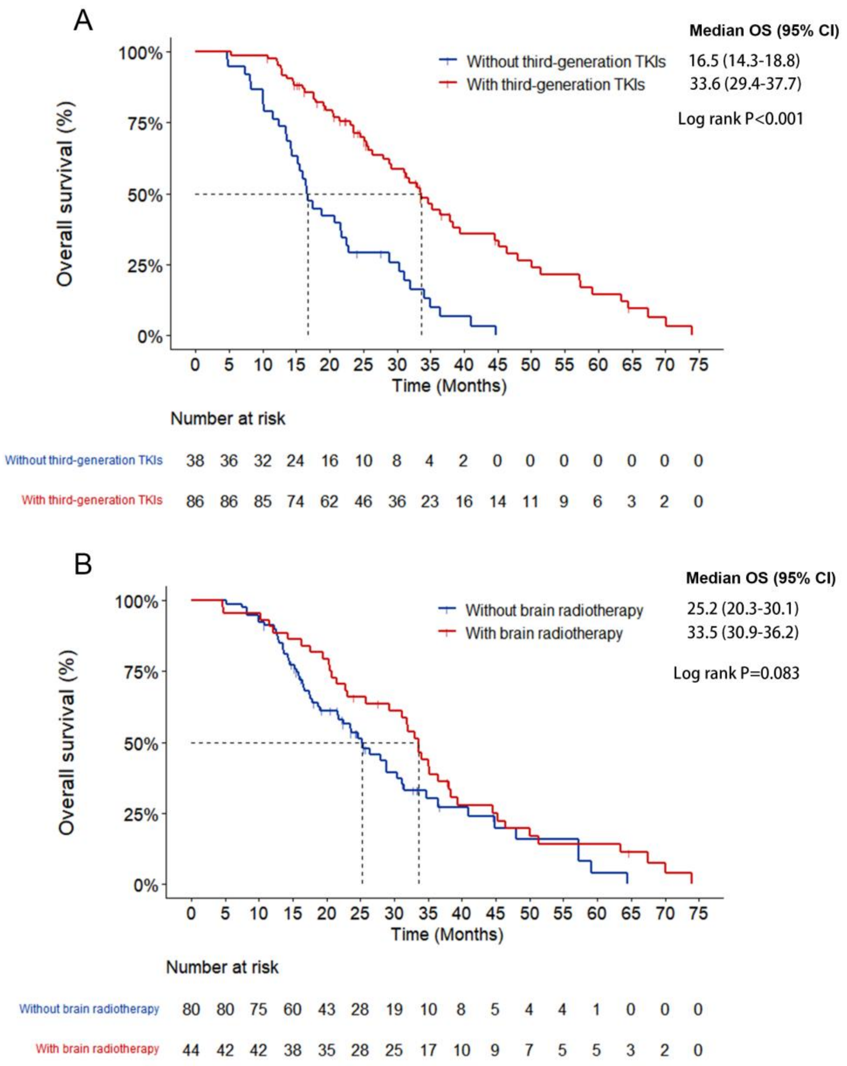

© 2023 Hou X et al. *JAMA Network Open.*

eFigure 7. Kaplan-Meier curves for overall survival incorporating the subsequent third-generation TKIs and brain radiotherapy upon progression.

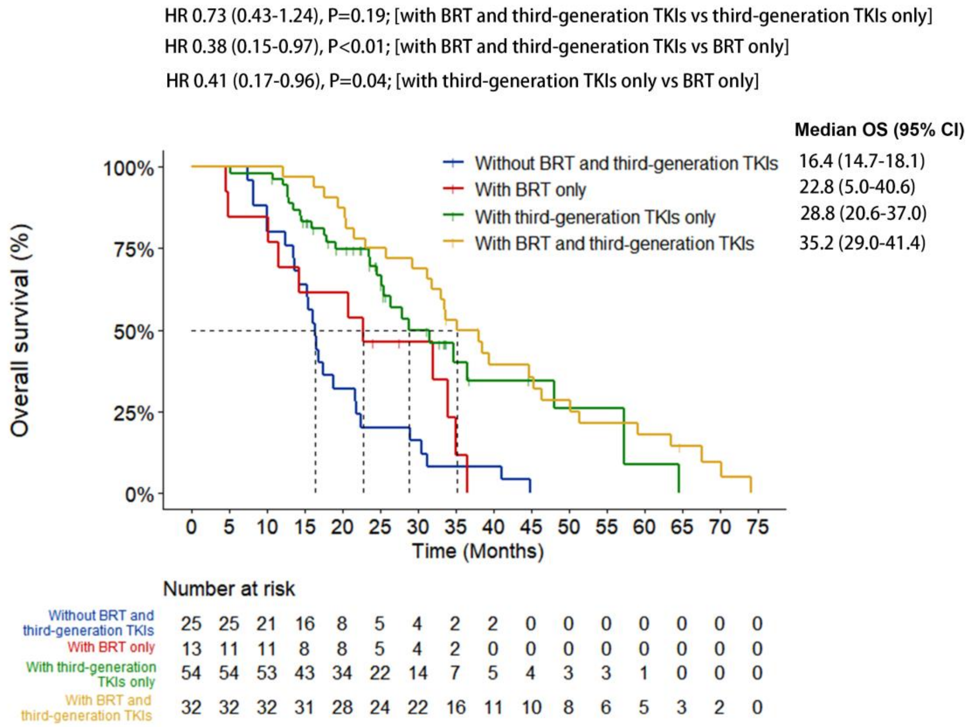

BRT, brain radiotherapy; TKIs, tyrosine kinase inhibitors. HR, hazard ratio.

© 2023 Hou X et al. *JAMA Network Open.*
