# Supplementary material for: Gefitinib Plus Chemotherapy vs Gefitinib Alone in Untreated EGFR-Mutant Non–Small Cell Lung Cancer in Patients With Brain Metastases: The GAP BRAIN Open-Label, Randomized, Multicenter, Phase 3 Study
Source: JAMA Netw Open. 2023 Feb 8;6(2):e2255050. doi: 10.1001/jamanetworkopen.2022.55050 (PMC9909498; doi:10.1001/jamanetworkopen.2022.55050)
Supplement: Supplement 3. — Data Sharing Statement [file jamanetwopen-e2255050-s003.pdf]

Data Sharing Statement

Hou. Gefitinib Plus Chemotherapy vs Gefitinib Alone in Untreated EGFR-Mutant Non-Small Cell Lung Cancer in Patients With Brain Metastases. *JAMA Netw Open*. Published February 08, 2023. doi:10.1001/jamanetworkopen.2022.55050

Data
Data available: Yes
Data types: Deidentified participant data
How to access data: data can be made available from the corresponding authors upon reasonable request, Dr Chen:chenlk@sysucc.org.cn.
When available: With publication

Supporting Documents
Document types: None

Additional Information
Who can access the data: data can be made available from the authors upon reasonable request.
Types of analyses: for any purpose
Mechanisms of data availability: with a signed data access agreement
Any additional restrictions: No additional restrictions.
